# Projection-dependent heterogeneity of cerebellar granule cell calcium responses

**DOI:** 10.1186/s13041-021-00773-y

**Published:** 2021-03-31

**Authors:** Jun Kyu Rhee, Heeyoun Park, Taegon Kim, Yukio Yamamoto, Keiko Tanaka-Yamamoto

**Affiliations:** 1 Center for Functional Connectomics, Brain Science Institute, Korea Institute of Science and Technology (KIST), Seoul, 02792 Republic of Korea; 2 Division of Bio-Medical Science and Technology, KIST School, Korea University of Science and Technology (UST), Seoul, 02792 Republic of Korea

**Keywords:** Cerebellar granule cells, Calcium imaging, AAV-driven labeling, Heterogeneity, Receptor distributions

## Abstract

Cerebellar granule cells (GCs) relay mossy fiber (MF) inputs to Purkinje cell dendrites via their axons, the parallel fibers (PFs), which are individually located at a given sublayer of the molecular layer (ML). Although a certain degree of heterogeneity among GCs has been recently reported, variability of GC responses to MF inputs has never been associated with their most notable structural variability, location of their projecting PFs in the ML. Here, we utilize an adeno-associated virus (AAV)-mediated labeling technique that enables us to categorize GCs according to the location of their PFs, and compare the Ca^2+^ responses to MF stimulations between three groups of GCs, consisting of either GCs having PFs at the deep (D-GCs), middle (M-GCs), or superficial (S-GCs) sublayer. Our structural analysis revealed that there was no correlation between position of GC soma in the GC layer and location of its PF in the ML, confirming that our AAV-mediated labeling was important to test the projection-dependent variability of the Ca^2+^ responses in GCs. We then found that the Ca^2+^ responses of D-GCs differed from those of M-GCs. Pharmacological experiments implied that the different Ca^2+^ responses were mainly attributable to varied distributions of GABA_A_ receptors (GABA_A_Rs) at the synaptic and extrasynaptic regions of GC dendrites. In addition to GABA_A_R distributions, amounts of extrasynaptic NMDA receptors appear to be also varied, because Ca^2+^ responses were different between D-GCs and M-GCs when glutamate spillover was enhanced. Whereas the Ca^2+^ responses of S-GCs were mostly equivalent to those of D-GCs and M-GCs, the blockade of GABA uptake resulted in larger Ca^2+^ responses in S-GCs compared with D-GCs and M-GCs, implying existence of mechanisms leading to more excitability in S-GCs with increased GABA release. Thus, this study reveals MF stimulation-mediated non-uniform Ca^2+^ responses in the cerebellar GCs associated with the location of their PFs in the ML, and raises a possibility that combination of inherent functional variability of GCs and their specific axonal projection contributes to the information processing through the GCs.

**Supplementary Information:**

The online version contains supplementary material available at 10.1186/s13041-021-00773-y.

## Introduction

Many studies have finely classified cortical neurons into larger number of neuronal types than traditionally identified [1–5]. Specifically, the classification by combination of physiological properties and network connectivity seems to have a great potential to directly associate each classified neuron type with functional significance. In fact, input- or projection-dependent physiological properties have been characterized in cortical or hippocampal pyramidal neurons [6–9].

The cerebellar cortex traditionally includes four major types of neurons. Among them, granule cells (GCs) are the only excitatory neurons and convey inputs from mossy fibers (MFs) to Purkinje cells, which are the sole output neurons of the cerebellar cortex [10, 11]. MFs and GCs synapse at the cerebellar glomeruli in the GC layer (GCL). A single glomerulus is made up of a single MF terminal, several tens of GC dendrites, and axons and dendrites of Golgi cells which innervate onto GC dendrites and receive inputs from MFs, respectively [12–14]. Through such synaptic connections in the glomeruli, MF stimulation triggers direct excitatory synaptic inputs by MF activation and indirect inhibitory synaptic inputs by Golgi cell activation to GCs [15–18].

The cerebellum has been characterized by a uniform cytoarchitecture and a conserved network structure. This perspective is gradually changing by the findings of non-uniform microcircuits and heterogeneous neuronal properties in tandem with the technological advances [19, 20]. Cerebellar GCs are small in size, constitute more than 50% of the neurons in the brain, and are closely packed in the GCL. Even though such characteristics of GCs make it difficult to investigate their non-uniformity, moderate yet evident heterogeneity has been reported regarding molecular expression (21), morphology [22] and physiological properties [23–27]. In addition, there is an obvious diversity in GC network structure, which is the location of their axons, parallel fibers (PFs), in the molecular layer (ML), and morphological and functional differences of PFs according to their locations have been reported. Studies using electron microscopy revealed that diameters of PFs located in the deeper ML were wider [26, 28–32]. Depending on the location of the PFs, the velocity of action potential propagation in PFs, as well as the processing of PF inputs by Purkinje cells was different [26]. Thus, the information processing through GCs is likely varied according to the locations of their PFs in the ML. Nevertheless, because the non-uniformity of GCs’ responses to MF inputs has never been established with respect to the locations of their PFs, the PF-location dependent GC information processing has not yet been entirely clarified.

In this study, we took advantage of a technique using an adeno-associated viral (AAV) vector that enables distinct labeling of GCs, whose PFs are located in the deep, middle, or superficial sublayer of the ML. We then compared the increase in intracellular calcium (Ca^2+^) concentration upon the MF stimulation among the three groups of many GCs by taking advantage of the acquisition speed and spatial resolution of the widefield fluorescence microscopy. We detected significant differences in the Ca^2+^ responses of GCs depending on the locations of their PFs, and further obtained results leading to a conclusion that the differences in receptor distributions appear to be responsible for the varied Ca^2+^ responses in the three groups of GCs. Thus, in addition to the morphological or functional differences of PFs themselves, the GCs’ responsiveness to MF inputs also differs according to the location of their PFs in the ML.

## Results

### Monitoring Ca^2+^ signals in different groups of GCs distinguished according to the location of their PFs

**Fig. 1:**
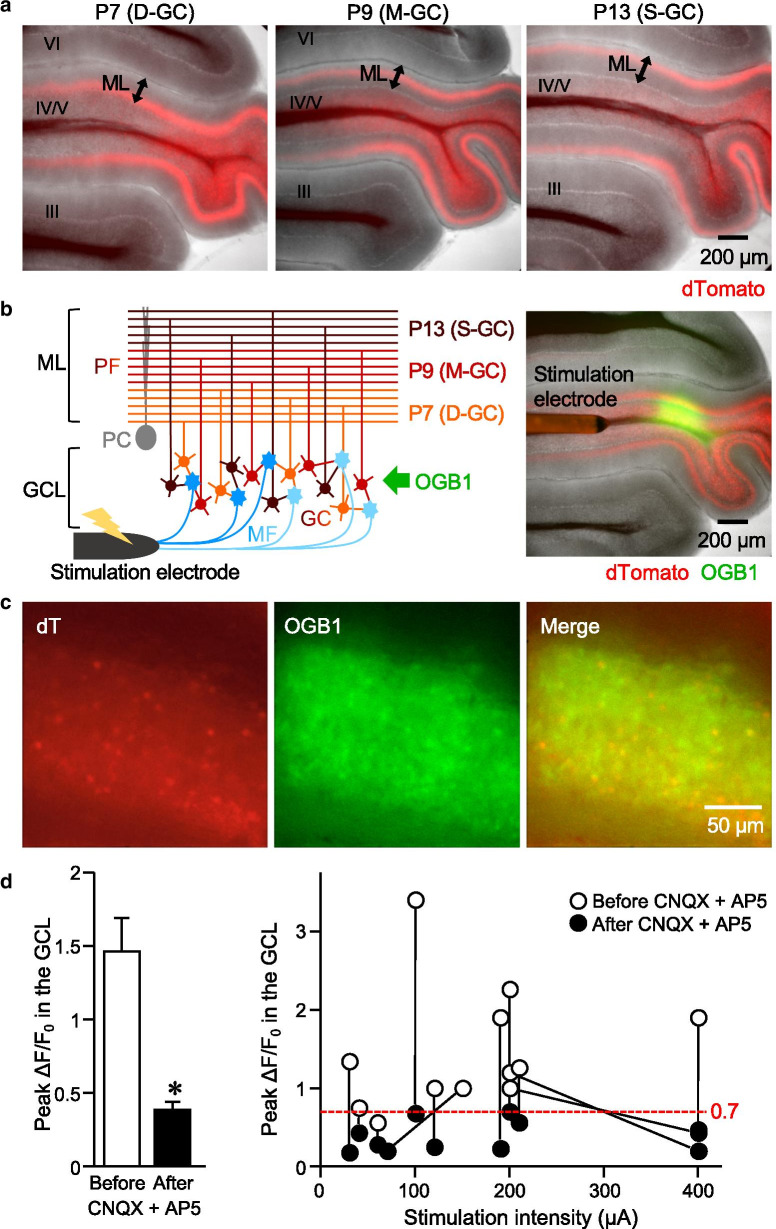
Detection of the Ca^2+^ responses in cerebellar slices labeled with AAV-GABRα6-dT. **a** Representative images of fresh cerebellar sagittal slices expressing dT (red) in D-GCs (left), M-GCs (middle), and S-GCs (right), which were achieved by the stereotaxic injection of AAV-GABRα6-dT at P7, P9, and P13, respectively. Note that the dT-positive PF bundles are at the deep, middle, and superficial sublayer of the ML. The roman numerals indicate the lobes. **b** Diagram (left) and an image (right) of experimental setting. OGB1 (green) was loaded in the GCL of cerebellar sagittal slices expressing dT (red), and the bipolar electrode was placed on the white matter to stimulate the MFs. **c** Magnified images of dT expression and OGB1 loading in GCs of a fresh cerebellar slice. **d** Peak ΔF/F_0_ in the whole GCL upon MF stimulation before and after the application of CNQX and AP5. The averaged values (left) and results obtained from individual slices (right) are shown (n = 12 slices, *p = 2.06 × 10^–4^, paired sample t-test). Error bars in this and subsequent figures indicate SEM. Exact *p* values for the datasets in this and subsequent figures are provided in Additional file 1: Table S2

To identify somas of GCs with PFs located within the deep (D-GCs), middle (M-GCs) or superficial (S-GCs) sublayer of the ML, we utilized the labeling method using AAV vectors with the minimum region of γ-aminobutyric acid (GABA) type A receptor (GABA_A_R) α6 subunit (GABRα6) promoter. Stereotaxic injection of AAV-GABRα6 at a proper timing during postnatal development is capable of triggering molecular expression in a group of GCs, whose PFs are bundled together at a certain sublayer of the ML [33]. In this study, we injected AAV-GABRα6 expressing dTomato (dT) into lobe IV/V of the mouse cerebellum at postnatal day 7 (P7), P9, or P13, which resulted in the labeling of D-GCs, M-GCs, or S-GCs with dT, respectively (Fig. 1a). Considering a massive number of GCs and possible heterogeneous responses in individual GCs, the key barrier that we have to overcome is the neutralization of differences due to the high variability caused by severe undersampling. Thus, we aimed to record Ca^2+^ signals from as many GCs as possible by utilizing widefield Ca^2+^ imaging. Widefield fluorescence imaging generally affords us both the high spatial resolution and the high-speed imaging, which potentially allow us to identify many GC somas and to accurately capture the time lapse profile of the Ca^2+^ signals in each GC soma. The widefield Ca^2+^ imaging combined with labeling by AAV-GABRα6 is therefore capable of simultaneous recording from many GCs and of direct comparison of responses in different groups of GCs. Ca^2+^ indicator dye, Oregon Green 488 BAPTA-1 (OGB1), was loaded in the GCL of fresh cerebellar sagittal slices that were obtained from mice subjected to the AAV injection (Fig. 1b). As a result, GCs showing variable intensity of dT signals were labeled with OGB1 (Fig. 1c). Upon the MF stimulation via a bipolar electrode placed on the white matter (Fig. 1b, 100 Hz, for 100 ms), the fluorescence intensity of OGB1 in the whole GCL increased (Fig. 1d), as seen in the positive values of normalized intensity change calculated as ΔF/F_0_ (see Methods). However, the increase was significantly reduced in the presence of inhibitors of AMPA- (AMPARs) and NMDA-type glutamate receptors (NMDARs), 6-Cyano-7-nitroquinoxaline-2,3-dione (CNQX) and D-AP5 (AP5) (Fig. 1d), indicating that the increase in the OGB1 intensity represented the increase in the Ca^2+^ signals mostly caused by MF-dependent synaptic excitation, but not by direct electrical stimulation onto GCs and other cells in the slice. When we looked at the results of individual slices before and after the application of CNQX and AP5, the peak ΔF/F_0_ after the application was always smaller than 0.7, while peak ΔF/F_0_ before the application was mostly larger than 0.7 (Fig. 1d, right). We therefore used results showing more than 0.7 of peak ΔF/F_0_ in the whole GCL for the analyses of this study, to minimize the influence of noise and to omit the results obtained from unhealthy slices.

**Fig. 2:**
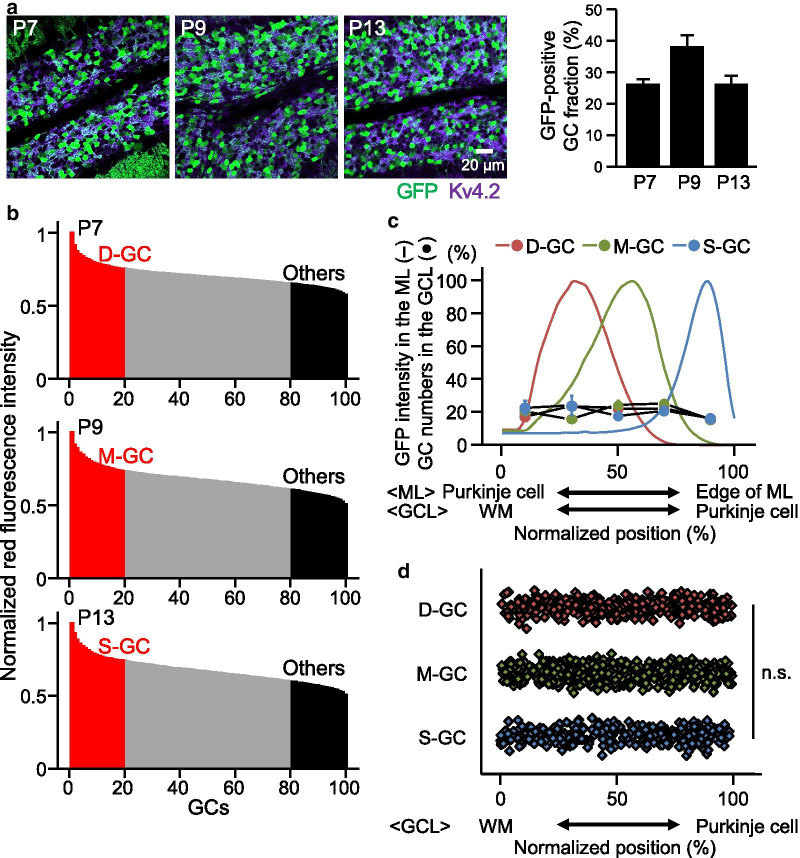
Percentages and distributions of GCs having expression triggered by AAV-GABRα6. **a** Confocal images (left) of fixed cerebellar sagittal slices having GFP expression (green) and stained with an antibody of Kv4.2 (purple). Bar graphs (right) show percentages of GFP-positive GCs among all GCs. Expression of GFP was triggered by the injection of AAV-GABRα6-GFP on the indicated postnatal days. **b** Examples of normalized red fluorescence intensity in 100 GCs selected in dT images of slices that were labeled by injecting at P7 (top), P9 (middle), or P13 (bottom). Twenty GCs with the highest intensity were considered dT-positive GCs and 20 GCs with the lowest intensity were considered other GCs. **c** Distribution of GFP intensity in the ML (colored lines) and GFP-positive GCs in the GCL (colored filled circles) along anterior–posterior axis (*N* = 4, 4, or 3 mice for D-GCs, M-GCs, or S-GCs, respectively). **d** Normalized positions of individual GFP-positive D-GCs, M-GCs, and S-GCs. No significant difference (n.s.) was detected in GC soma distribution between the three groups of GCs (*p* = 0.574, ANOVA). The x-axes in **c** and **d** show normalized position from the edge of Purkinje cell somas (0) to edge of ML (100), or from boundary between WM and GCL (0) to edge of Purkinje cell somas (100)

We next tried to identify individual GCs and look at their Ca^2+^ responses. Because GCs are tightly packed in the GCL, individual GCs were hardly distinguishable using bright field images. Instead, we selected 100 GCs from a single image (210 × 210 μm) of one side of GCL by utilizing red fluorescence images, which are presumably composed of not only dT fluorescence but also autofluorescence. To estimate ratios of GCs containing genuine dT fluorescence among all GCs, fixed slices were obtained from mice subjected to the injection of AAV-GABRα6 expressing GFP at P7, P9, or P13, and were stained with an antibody of Kv4.2 voltage-gated potassium channels, a marker of GC membranes. We then found that approximately 25–35% of GCs were GFP positive (Fig. 2a). For analyzing the Ca^2+^ responses in fresh slices, we therefore considered 20 GCs showing the highest fluorescence intensity among the selected 100 GCs to be the dT-positive GCs, representing D-GCs, M-GCs, and S-GCs labeled by injecting at P7, P9 and P13, respectively. To minimize the possibility of misidentifying genuine dT-positive GCs as “other” GCs, GCs other than D-GCs, M-GCs, and S-GCs, 20 GCs showing the weakest red signals were defined to be the other GCs (Fig. 2b). In the fixed slices, the GFP-positive GC somas were scattered in the GCL (Fig. 2a), unlike their GFP-positive PFs which clustered to a specific sublayer within the ML. To confirm this, we analyzed the locations of GFP-positive GC somas along the anterior–posterior axis in the fixed slices. Even though GFP-positive PFs were clustered in the ML, as seen in the bell shape of GFP intensity distribution across the ML (Fig. 2c), GFP-positive GC somas were evenly distributed along the anterior–posterior axis of the GCL (Fig. 2c, d). Thus, our AAV-driven labeling method was well-suited to identify specific GCs according to the locations of their PFs, which led to the analysis of PF location dependent Ca^2+^ responses.

**Fig. 3:**
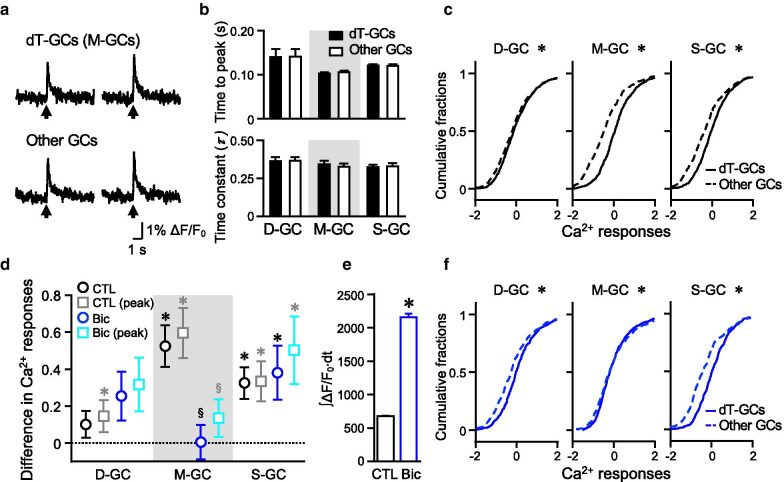
Difference in the Ca^2+^ increase in D-GCs, M-GCs, and S-GCs. **a** Examples of the time course of Ca^2+^ responses in individual dT-positive M-GCs and other GCs. Arrows indicate the timing of MF stimulation. **b** Comparison of time to peak (top) and decay time constants (bottom) of the Ca^2+^ responses between the dT-positive GCs (D-GCs, M-GCs or S-GCs) and their other GCs in control (n = 53, 31, or 53 slices for D-GCs, M-GCs, or S-GCs, respectively). **c** The cumulative distributions of the Ca^2+^ responses in D-GCs, M-GCs, and S-GCs (solid lines; n = 1060, 620, or 1060 cells for D-GCs, M-GCs, or S-GCs, respectively) compared with the distributions in their other GCs (dotted lines; n = 1060, 620, or 1060 cells for D-GCs, M-GCs, or S-GCs, respectively) (*p < 0.05, KS test). **d** Difference in Ca^2+^ responses between the dT-positive GCs (D-GCs, M-GCs, or S-GCs) and their other GCs in control (black open circles; n = 53, 31, or 53 slices for D-GCs, M-GCs, or S-GCs, respectively) or in the presence of bicuculline (blue open circles; n = 27, 39, or 26 slices for D-GCs, M-GCs, or S-GCs, respectively) (for the comparison with 0, *p < 0.05, one-sample Wilcoxon signed rank test; for comparison between control and bicuculline, ^§^*p* < 0.05, one-way ANOVA followed by the Fisher test). Gray and cyan squares show data analyzed by using the standardized peak ΔF/F_0_, instead of the standardized ∫ΔF/F_0_∙dt. **e** Increase in overall Ca^2+^ increase upon MF stimulation by the application of bicuculline (n = 5472 GCs for control, n = 3677 GCs for bicuculline, *p = 0, Mann–Whitney test). **f** The cumulative distributions of the Ca^2+^ responses in the presence of bicuculline. The distribution is compared between D-GCs, M-GCs, or S-GCs (solid lines; n = 540, 780, or 520 cells for D-GCs, M-GCs, or S-GCs, respectively) and their other GCs (dotted lines; n = 540, 780, or 520 cells for D-GCs, M-GCs, or S-GCs, respectively) (*p < 0.05, KS test). Calculation of the Ca^2+^ responses in this and the subsequent figures were described in Methods

An increase in Ca^2+^ signals was observed upon MF stimulation in both dT-positive and other GCs. Example traces of changes in ΔF/F_0_ over time were hardly distinguishable between the dT-positive and the other GCs, as seen in Fig. 3a. Quantitative analysis showed no significant differences in time to peak and decay time constants between any groups of dT-positive GCs and other GCs (Fig. 3b). However, plotting the cumulative distribution of the amount of Ca^2+^ responses, which was quantified as standardized ∫ΔF/F_0_∙dt (z-scores of Ca^2+^ responses, which we mainly use to examine “Ca^2+^ responses”; see Methods), from a large number of GCs suggested that the overall Ca^2+^ increase was larger in M-GCs or S-GCs compared to their respective other GCs, as indicated by the rightward shifts in the cumulative distribution curves of M-GCs and S-GCs compared to those of their other GCs (Fig. 3c). The Kolmogorov–Smirnov test (KS test) showed significant differences between not only M-GCs, S-GCs, but also D-GCs, and their other GCs. The larger responses in M-GCs and S-GCs were further confirmed by calculating the differences in the Ca^2+^ responses between each group of GCs (D-, M-, or S-GCs) and other GCs in individual slices. As seen in Fig. 3d (black open circles), M-GCs and S-GCs, but not D-GCs, showed significantly positive differences. When we analyzed using the standardized peak ΔF/F_0_ (gray squares in Fig. 3d) instead of the standardized ∫ΔF/F_0_∙dt, similarly larger responses in M-GCs and S-GCs were observed. Two-way ANOVA (*p* = 0.642) confirmed no significant difference between the comparisons performed using the standardized ∫ΔF/F_0_∙dt (black circles in Fig. 3d) and the standardized peak ΔF/F_0_ (gray squares in Fig. 3d). Although standardized values (z-scores) are mainly used in this study to limit the influence of slice to slice variability, unstandardized ∫ΔF/F_0_∙dt and unstandardized peak ΔF/F_0_ showed consistent results in control conditions (Additional file 1: Fig. S1). Based on the difference in ∫ΔF/F_0_∙dt and total amounts of ∫ΔF/F_0_∙dt, the size of large responses in M-GCs and S-GCs could be estimated as 16.3% and 13.7% of the total Ca^2+^ increase, respectively (Additional file 1: Table S1). The results so far suggest that M-GCs or S-GCs include GCs exhibiting larger Ca^2+^ increase upon MF stimulation.

Although the widefield Ca^2+^ imaging is appropriate for measuring the Ca^2+^ responses from many GCs at once, it has a weakness in that OGB1 signals of individual GCs presumably include contaminating signals from out-of-focus GCs. Because the contaminating signals would similarly affect the dT-positive GCs and the other GCs, they may possibly obscure the difference in the Ca^2+^ responses between two GC groups. To minimize the effects of out-of-focus signals, we compared the Ca^2+^ responses in selected pairs of neighboring dT-positive GCs and other GCs, both of which were in focus, whereas such selection of the GC pairs considerably harmed the sample size (31–53 GC pairs, instead of 620–1060 GC pairs). We detected no significant difference in the analysis for D-GCs (Additional file 1: Fig. S2a), suggesting that no difference between D-GCs and other GCs in the original analysis (Fig. 3d) is not due to the interference from the contaminating signals. We also detected no significant difference in the analysis of M-GCs or S-GCs with their neighboring other GCs (Additional file 1: Fig. S2a). We assumed that this may be because of the small number of analyzed GCs. To confirm this assumption, we compared the Ca^2+^ responses between certain numbers (N) of randomly sampled pairs of the dT-positive GCs and the other GCs (Additional file 1: Fig. S2b). The variability inversely correlated with N. Specifically, when N was as small as the analysis of neighboring GCs (n = 1 pair sampled per slice), the difference in Ca^2+^ responses was not significant in most cases, whereas the difference was significant for M-GCs and S-GCs when N was large (Additional file 1: Fig. S2c, d). Thus, our strategy of measuring as many GCs as possible seems appropriate to detect the small differences in the Ca^2+^ responses between the different groups of GCs.

### Contribution of GABA_A_ receptors on the different Ca^2+^ responses

Because GC activity can be controlled by GABA released from Golgi cells [34], we tested the effects of GABA_A_ receptors (GABA_A_Rs) on the Ca^2+^ responses of the GCs. Although we sometimes reduced the intensity of electrical MF stimulation to avoid saturating the Ca^2+^ responses, overall Ca^2+^ increase (∫ΔF/F_0_∙dt) was higher in the presence of bicuculline, a GABA_A_R antagonist, than in control (Fig. 3e), indicating that GABA_A_R-mediated inhibition indeed regulates GC activity. Our analysis of difference in the Ca^2+^ responses demonstrated that the Ca^2+^ responses in S-GCs, but not D-GCs or M-GCs, were larger than other GCs, as shown by significantly positive values in the presence of bicuculline (Fig. 3d, blue open circles). Consequently, in the presence of bicuculline, the difference in Ca^2+^ responses between M-GCs and other GCs was significantly smaller than the control condition, while the differences between D-GCs or S-GCs and other GCs were comparable to control condition. As was the case in control, results obtained by using the standardized peak ΔF/F_0_ (cyan squares in Fig. 3d) were comparable to the results obtained by using the standardized ∫ΔF/F_0_∙dt (*p* = 0.305, two-way ANOVA). Consistently, time to peak and decay time constants of the Ca^2+^ responses are equivalent between each group of GCs and other GCs (Additional file 1: Fig. S3a). We therefore used the standardized ∫ΔF/F_0_∙dt for the following analyses in this study when comparing the Ca^2+^ responses between GC groups. The larger Ca^2+^ responses in S-GCs were also apparent in the rightward shift of the cumulative distribution curve (Fig. 3f), although the KS test showed significant differences between not only S-GCs, but also D-GCs or M-GCs, and their other GCs. Different effects of bicuculline on the difference in the Ca^2+^ responses suggest distinct involvement of GABA_A_Rs in regulating the Ca^2+^ responses among the different groups of GCs. We note that results are essentially same, when other GCs were defined by 60 GCs instead of 20 GCs showing the weakest red signals (Additional file 1: Fig. S3b), implying that the selection scheme of the other GCs does not have a major impact on the results.

### Direct comparison between two groups of GCs

**Fig. 4:**
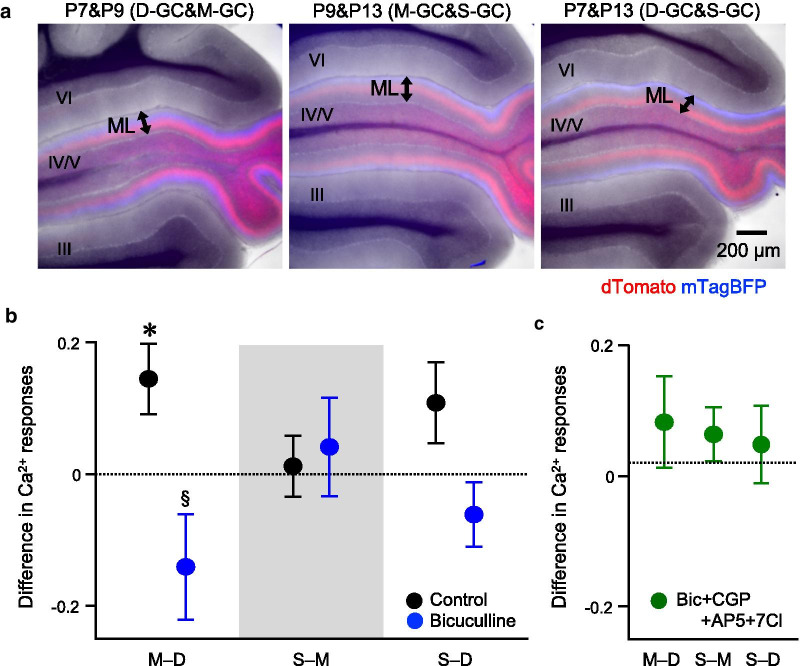
Direct comparison of Ca^2+^ increase by double labeling. **a** Images of fresh cerebellar sagittal slices expressing dT (red) and mTB (blue) in different groups of GCs. Note that the dT- and mTB-positive PF bundles are located at different sublayers of the ML. The roman numerals indicate the lobes. **b**, **c** Difference in Ca^2+^ responses between the two groups of GCs in control (**b**, black, n = 32, 33, or 27 slices for M–D, S–M, or S–D, respectively), in the presence of bicuculline (**b**, blue, n = 17, 18, or 10 slices for M–D, S–M, or S–D, respectively), or the combination of four antagonists (**c**, green, n = 12, 18, or 19 slices for M–D, S–M, or S–D, respectively), bicuculline (Bic), CGP, AP5, and 7Cl (for the comparison with 0, *p < 0.05, one-sample Wilcoxon signed rank test; for comparison between control and bicuculline, ^§^*p* < 0.05, one-way ANOVA followed by the Fisher test)

Our analysis of the Ca^2+^ responses in GCs labeled by injecting AAV-GABRα6-dT demonstrated that the differences in the Ca^2+^ responses varied among D-GCs, M-GCs, and S-GCs in control or in the presence of bicuculline. Although the results suggest that the GC Ca^2+^ responses vary according to its PF location, observation of only positive differences (Fig. 3d) raised the possibility of underestimated Ca^2+^ responses in other GCs that were selected by autofluorescence, resulting in overestimated size of differences. To resolve the issue, we performed direct pairwise comparisons between the GC groups by labeling two groups of GCs with two different fluorescence molecules, dT and mTagBFP2 (mTB). For these experiments, AAV-GABRα6-dT and AAV-GABRα6-mTB were injected into lobe IV/V of the cerebellum at two different time points. The injection at P7 and P9, P9 and P13, or P7 and P13 resulted in the labeling of D-GCs and M-GCs, M-GCs and S-GCs, or D-GCs and S-GCs, respectively (Fig. 4a). As was the case in single injections, GCL of the fresh cerebellar slices were loaded with OGB1 to measure the Ca^2+^ responses. We then compared the Ca^2+^ responses between 20 dT-positive GCs and 20 mTB-positive GCs by calculating the difference in the Ca^2+^ responses (e.g., M–D, subtracting the average of Ca^2+^ responses in D-GCs from that in M-GCs). Consequently, the positive value obtained by this calculation denotes a larger Ca^2+^ response in the former group than the latter group of GCs. Thus, this analysis allowed us to make direct pairwise comparisons of the Ca^2+^ responses among the three groups of GCs. In control, the Ca^2+^ responses in M-GCs were larger than D-GCs, whereas the Ca^2+^ responses in S-GCs were equivalent to those in M-GCs and D-GCs (Fig. 4b, black filled circles). Effect size of the difference in Ca^2+^ responses between M-GCs and D-GCs can be estimated as approximately 3.5% of the total Ca^2+^ increase (Additional file 1: Table S1). In the presence of bicuculline, there were no significant differences between the three groups of GCs (Fig. 4b, blue filled circles). Compared with the control condition, bicuculline caused a significant reduction in the difference in the Ca^2+^ responses between D-GCs and M-GCs. This indicates that GABA_A_Rs are the source producing the difference in Ca^2+^ responses between M-GCs and D-GCs. Considering the results of the double injection, the order of strength in the Ca^2+^ responses is M-GCs > D-GCs in control, whereas there is no clear order in the presence of bicuculline.

In addition to bicuculline, we also added a GABA_B_ receptor (GABA_B_R) antagonist, CGP35348 (CGP), together with two NMDAR antagonists, 7-Chlorokynurenic acid (7Cl) and AP5. The differences in the Ca^2+^ responses were negligible in all comparisons (Fig. 4c). The results of combination of four antagonists indicate that the remaining components, such as AMPARs or metabotropic glutamate receptors (mGluRs), barely contribute to generating the differences in the Ca^2+^ responses among the three groups of GCs.

### Mechanisms producing differences in Ca^2+^ responses between D-GCs and M-GCs

**Fig. 5:**
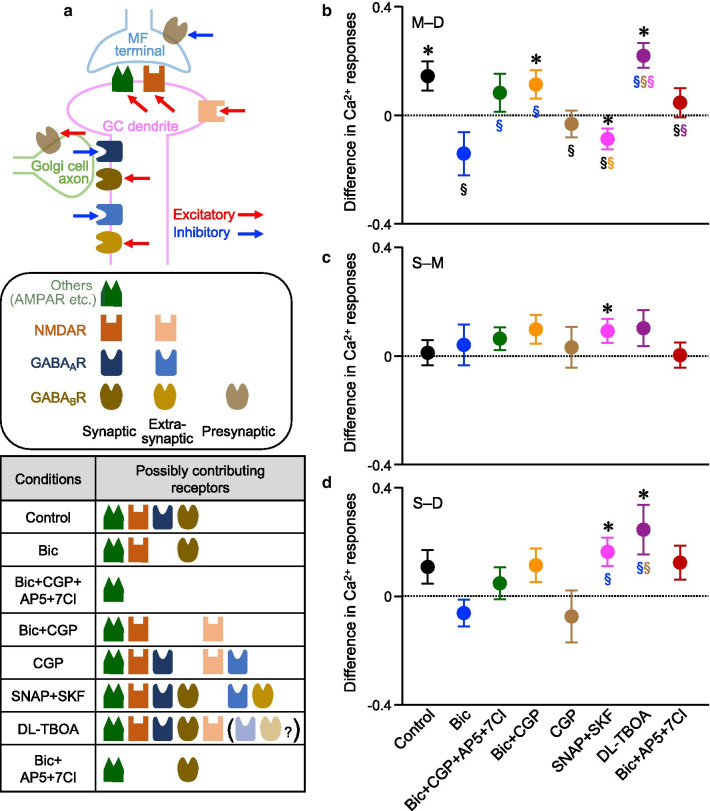
Comparison of the Ca^2+^ increase in different conditions. **a** Schematic diagram of excitatory and inhibitory receptor components that are considered in this study. The table at the bottom indicates putative components that are activated in the given condition. Individual components are explained in the center. **b**-**d** Difference in Ca^2+^ responses between two groups of GCs in several conditions indicated on the x-axis. Comparison was made between M-GCs and D-GCs (**b**), S-GCs and M-GCs (**c**), or S-GCs and D-GCs (**d**) (for the comparison with 0, *p < 0.05, one-sample Wilcoxon signed rank test; for comparison in the different conditions, ^§^*p* < 0.05, one-way ANOVA followed by the Fisher test, color symbols indicate the comparison with conditions for which the results are shown in that color). For the direct comparison, results shown in Fig. 4b, c are also included. Numbers of slices used are as follows: n = 19, 18, or 18 for M–D, S–M, or S–D, respectively (Bic + CGP); n = 26, 13, or 7 for M–D, S–M, or S–D, respectively (CGP); n = 25, 14, or 14 for M–D, S–M, or S–D, respectively (SNAP + SKF); n = 19, 12, or 10 for M–D, S–M, or S–D, respectively (DL-TBOA); n = 28, 31,or 23 for M–D, S–M, or S–D, respectively (Bic + AP5 + 7Cl)

We further investigated whether the Ca^2+^ responses can be varied between the different GC groups in other situations by using inhibitors, to dissect the underlying mechanisms to generate the difference in Ca^2+^ responses between GC groups. Because the difference in Ca^2+^ responses was observed between D-GCs and M-GCs in control, we first focused on comparing responses between D-GCs and M-GCs (M–D). Schematic diagram of excitatory and inhibitory receptor components considered in this study are shown in Fig. 5a. Because the Ca^2+^ responses were larger in M-GCs than D-GCs in control (Fig. 5b, black filled circle), but were equivalent between D-GCs and M-GCs in the presence of bicuculline (Fig. 5b, blue filled circle), M-GCs and D-GCs receive similar excitatory MF inputs, while M-GCs receive weaker GABA_A_R-mediated inhibition than D-GCs. The equivalent responses in the presence of four antagonists (Fig. 5b, green filled circle) confirmed that excitatory factors other than NMDARs and GABA_B_Rs are similar between D-GCs and M-GCs, although the effects of GABA_B_Rs are presumably complex involving both inhibitory and excitatory pathways [34]. To see whether the NMDAR-mediated excitation is similar between D-GCs and M-GCs, we tested the Ca^2+^ responses without the influence of GABA_A_Rs and GABA_B_Rs by applying bicuculline and CGP. Unexpectedly, the Ca^2+^ responses in M-GCs were larger than D-GCs (Fig. 5b, orange filled circle). Activation of presynaptic GABA_B_Rs on MF terminals is known to reduce glutamate release [35, 36], so that inhibition of GABA_B_Rs by CGP may result in enhancement of glutamate release and consequent activation of extrasynaptic NMDARs [15, 37]. Thus, this result suggests that more extrasynaptic NMDARs present on M-GCs may contribute to the larger Ca^2+^ responses in M-GCs than D-GCs, when glutamate release is enhanced.

Under the assumption that more extrasynaptic NMDARs in M-GCs could be activated by the inhibition of GABA_B_Rs, we expected to see even larger Ca^2+^ responses in M-GCs in the presence of only CGP without bicuculline. Contrary to the expectation, the Ca^2+^ responses in M-GCs were equivalent to those in D-GCs (Fig. 5b, brown filled circle). Considering that the presynaptic GABA_B_Rs are also known to exist on Golgi cell terminals and their activation leads to reduced GABA release [38], our results suggest that more extrasynaptic GABA_A_Rs present on M-GCs would reduce the Ca^2+^ responses of M-GCs, when excessive GABA is released upon the inhibition of GABA_B_Rs. To test this idea, we used (S)-SNAP 5114 (SNAP) and SKF 89976A (SKF), inhibitors of GABA transporter type 3 and 1, respectively, that prevent GABA reuptake to allow stronger activation of extrasynaptic GABA_A_Rs. The combination of GABA uptake inhibitors made the Ca^2+^ responses in M-GCs smaller than D-GCs, as seen in the significantly negative value of the difference in the Ca^2+^ responses (Fig. 5b, magenta filled circle), indicating that there are more extrasynaptic GABA_A_Rs in M-GCs than D-GCs.

Similar to the experiments using GABA uptake inhibitors, we also tested the idea of more extrasynaptic NMDARs in M-GCs by using DL-threo-β-Benzyloxyaspartic acid (DL-TBOA), a glutamate transporter inhibitor. Application of DL-TBOA could lead to strong activation of extrasynaptic NMDARs, and therefore was expected to result in larger difference in the Ca^2+^ responses between M-GCs and D-GCs than control. Unexpectedly, the difference in the presence of DL-TBOA was equivalent to control (Fig. 5b, purple filled circle compared with black filled circle). This may lead to another idea that the difference in Ca^2+^ responses between D-GCs and M-GCs in the presence of bicuculline and CGP might be due to the varied degree of glutamate spillover rather than the amounts of extrasynaptic NMDARs. However, it is less likely, because the decay time constants of the Ca^2+^ responses are not different between D-GCs and M-GCs (Additional file 1: Fig. S4). Alternatively, considering that the Golgi cells could be more active in the presence of DL-TBOA [39], a partial activation of extrasynaptic GABA_A_Rs possibly compensates the excitation by extrasynaptic NMDARs. Taken together, according to our experiments using several inhibitors, the numbers of GABA_A_Rs that are activated in the absence of reuptake inhibitors, referred to as synaptic GABA_A_Rs, appear to be less in M-GCs than D-GCs, yet the numbers of extrasynaptic GABA_A_Rs and probably extrasynaptic NMDARs appear to be more in M-GCs than D-GCs.

In addition to the presynaptic GABA_B_Rs on MF terminals and Golgi cells, it has been shown that the postsynaptic GABA_B_Rs are also present on GCs [40]. We examined the impact of GABA_B_Rs on the difference of Ca^2+^ responses between M-GCs and D-GCs by applying bicuculline, AP5 and 7Cl, which block GABA_A_Rs and NMDARs. In this condition, the Ca^2+^ increase in M-GCs was equivalent to that in D-GCs (Fig. 5b, red filled circle). Because the difference in Ca^2+^ responses between M-GCs and D-GCs was negligible with or without CGP (Fig. 5b, green and red filled circles), the influence of postsynaptic GABA_B_Rs seems to be similar between M-GCs and D-GCs.

### Comparison of components contributing to Ca^2+^ responses among the three groups of GCs

**Fig. 6:**
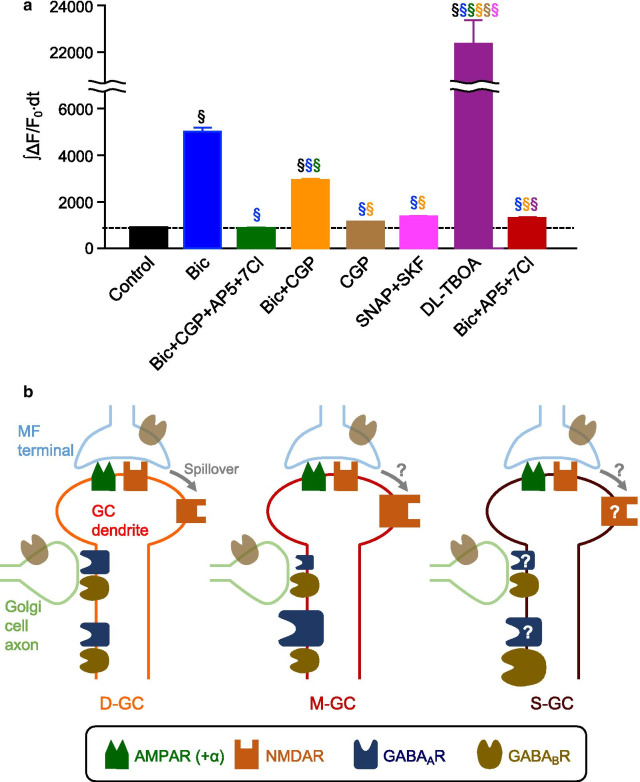
Mechanisms underlying different Ca^2+^ responses. **a** Overall Ca^2+^ increase, represented by ∫ΔF/F_0_∙dt, upon MF stimulation in the presence of inhibitors (^§^*p* < 0.05, one-way ANOVA followed by the Fisher test, color symbols indicate the comparison with conditions for which the results are shown in that color). Numbers of cells analyzed are as follows: n = 3680 (control); n = 1800 (Bic); n = 1960 (Bic + CGP + AP5 + 7Cl); n = 2200 (Bic + CGP); n = 1840 (CGP); n = 2120 (SNAP + SKF); n = 1640 (DL-TBOA); n = 3240 (Bic + AP5 + 7Cl). **b** Diagram of possible mechanisms that produce differences in MF-dependent Ca^2+^ increase in D-GCs, M-GCs and S-GCs

We also explored the difference in Ca^2+^ responses between S-GCs and M-GCs (S–M, Fig. 5c), and between S-GCs and D-GCs (S–D, Fig. 5d), and found that the differences for both S–M and S–D were mostly negligible. There was no significant difference in the control or in the presence of bicuculline alone, all four antagonists, bicuculline and CGP, CGP alone, or three antagonists of bicuculline, AP5, and 7Cl. On the other hand, we detected a difference in Ca^2+^ responses for both S–M and S–D in the presence of SNAP and SKF, as seen in the significantly positive values (Fig. 5c, d, magenta filled circle). The results indicate that S-GCs have mechanisms producing larger Ca^2+^ responses when an excessive amount of GABA is released. One possible mechanism serving such functions without strongly affecting the Ca^2+^ responses in other conditions tested in this study would be the extrasynaptic GABA_B_Rs that are presumably activated by an excessive amount of GABA release. Considering that the postsynaptic GABA_B_Rs in GCs could have pro-excitatory effect via reduction of Golgi cell-mediated IPSCs or inhibition of constitutive inward rectifier currents [40, 41], more extrasynaptic GABA_B_Rs may contribute to the larger Ca^2+^ increase observed in S-GCs in the presence of SNAP and SKF. To see whether we could detect pro-excitatory effects of GABA_B_Rs in our analysis, we compared the magnitude of ∫ΔF/F_0_∙dt in all GCs in all the different conditions of antagonists tested (Fig. 6a). The comparison in Ca^2+^ increase with and without CGP in control (comparison between black and brown in Fig. 6a) or in addition to bicuculline, AP5, and 7Cl (comparison between green and red in Fig. 6a) did not verify any effects of GABA_B_R on Ca^2+^ responses. On the other hand, Ca^2+^ increase observed in the presence of only bicuculline was reduced by additional application of CGP (comparison between blue and orange in Fig. 6a). Because blocking presynaptic GABA_B_R on MF terminals is supposed to enhance glutamate release and induce a larger Ca^2+^ response, not a smaller response, this result suggests the presence of pro-excitatory postsynaptic GABA_B_Rs. Thus, larger numbers of extrasynaptic GABA_B_Rs with pro-excitatory actions seem to be a good candidate mechanism producing the larger Ca^2+^ responses observed upon the excessive release of GABA in S-GCs. The larger numbers of extrasynaptic GABA_B_Rs may also contribute to the larger Ca^2+^ responses in S-GCs than D-GCs in the presence of DL-TBOA (Fig. 5d, purple filled circle), which possibly leads to more GABA release in addition to the glutamate spillover, as described above.

## Discussion

Although cerebellar GCs seem to be more homogeneous compared to other types of neurons, heterogeneity in structural and functional properties of GCs has been reported [21–27]. In this study, we categorized GCs based on the apparent structural variability, location of their PF projections within the ML, by utilizing an AAV-mediated labeling method, and compared the MF stimulation-dependent Ca^2+^ responses among the categorized GCs. The Ca^2+^ responses of the GCs could vary according to the location of their PF projection within the ML, implying that there are structurally associated functional differences among the GCs. In addition, our experiments using several combinations of inhibitors indicated that D-GCs differed from M-GCs mainly in the distribution of GABA_A_Rs at the synaptic and extrasynaptic regions and in mechanisms related to glutamate spillover, presumably amounts of extrasynaptic NMDARs (Fig. 6b). Despite the larger Ca^2+^ responses in M-GCs than D-GCs in control or in the presence of bicuculline and CGP, the Ca^2+^ responses in S-GCs were equivalent to both M-GCs and D-GCs in those conditions. Although mechanisms reconciling the superficially contradicting results are not currently clear, one simple idea is that components differing between D-GCs and M-GCs, such as distribution of GABA_A_Rs, might be at an intermediate level in S-GCs (Fig. 6b). On the other hand, we found a clear difference in Ca^2+^ responses of S-GCs compared with D-GCs and M-GCs, in the mechanisms responding to the excessive amounts of GABA release (Fig. 6b). This study suggests that the synaptic and extrasynaptic receptor compositions in cerebellar GCs can be organized based on their network structures, and proposes that structurally associated functional heterogeneity has to be considered to understand how GCs process and present the MF input to the Purkinje cell dendritic tree in the ML.

A very large number of GCs gradually differentiate during postnatal development, and PFs of early-born GCs are located in the deep sublayer, while PFs of late-born GCs are located in the superficial sublayer of the ML [42]. Our analysis of GFP-positive GCs vastly labeled by the injection of AAV-GABRα6-GFP reveals that GFP-positive GC somas were not clustered along anterior–posterior axis of the GCL, and that apparent unorganized distribution of GC somas was not different among D-GCs, M-GCs, and S-GCs, whose PFs were clearly bundled in the deep, middle, and superficial sublayers of ML, respectively. Although a recent study analyzing DiI-labeled GCs showed a correlation between the position of GC somas in the GCL and their PFs in the ML [26], our results are consistent with the three other studies that analyzed the distributions of GCs and PFs labeled by mosaic analysis with double marker, in vivo electroporation, or loading of Ca^2+^ indicator into a small cluster of GCs [43–45]. Because of the uncorrelated nature of positions of GC somas in the GCL and their PFs in the ML, labeling GCs according to the location of their PFs by our AAV-GABRα6 method was critical in analyzing PF location-associated functional variability in GCs. This labeling of GCs might also make it possible to detect the variability by patch clamp analysis that have been commonly used to see the MF simulation-dependent responses in GCs [27, 46, 47], or by higher spatial resolution imaging, such as confocal or two-photon imaging. These techniques provide us with more precise signals from the individual GCs. However, issues of sufficient sample size with proper temporal resolution may need to be dealt with, as seen in our analysis of neighboring GCs and computationally selected random GC pairs (Additional file 1: Fig. S2). Despite the contamination from the scattered light that is characteristic of the widefield fluorescence microscopy, our analysis of Ca^2+^ imaging was effective in detecting moderate variability; large imaging window with a high spatial resolution allowed us to work with a large sample size, high speed imaging allowed us to accurately capture the time lapse profile of the Ca^2+^ signals in each GC soma identified, and comparison of the GC groups within the same slices could overcome possible issues arising from biological noise or the small sample size artifact. On the other hand, there could be limitations in our methods. Due to the contaminating signals, we may have missed some significant variations in Ca^2+^ responses under some conditions. There is also a possibility that our labeling method may not be fully sufficient to detect clear variability in Ca^2+^ responses. If finer subcategorization of GCs according to their PF locations could be achieved, one of the finer groups might display clearly different properties in MF stimulation-evoked Ca^2+^ responses. Further efforts to develop a subgroup labeling technology of GCs seem to be necessary.

In the control condition of our experiments, D-GCs were less active compared to M-GCs. When the GABA_A_Rs were inhibited, responses in D-GCs became similar to M-GCs, whereas the order of response strength reverted by the enhanced glutamate release with inhibition of GABA_A_Rs. These results suggest that the activity balance between D-GCs and M-GCs could be altered by the situations affecting the overall excitation-inhibition (E-I) balance in the GCL. A recent study reported that acetylcholine regulates the balance of synaptic excitation and inhibition in the GCL and consequently GC excitability [48]. Anatomical studies showed the presence of cholinergic projections into the cerebellum by the observation of choline acetyltransferase-positive MF-like terminals or thin beaded fibers originating from diverse sources [49, 50]. Considering that acetylcholine release could be enhanced in the visual cortex by attention-engaging visual stimulation [51], some types of sensory stimuli may similarly enhance acetylcholine release in the cerebellum, although acetylcholine regulation in the cerebellum is totally unknown. In addition to acetylcholine, other pathways modulating Golgi cell activity would also presumably regulate the E-I balance in GCs, because Golgi cells are the source of the synaptic inhibition to GCs. GABAergic and glycinergic inhibitory inputs have been reported in Golgi cells. Although the origin and regulation of these inhibitory inputs to Golgi cells seem to be complex and are not yet clarified [52–57], it was suggested that the major inhibitory inputs to Golgi cells arose from outside of the cerebellar cortex [55]. This leads to a possibility that the inhibitory inputs to Golgi cells can be independently regulated from the excitatory inputs of MFs, and such independent regulation of the excitatory and inhibitory inputs to GCs would allow alteration of the E-I balance in GCs. Because MF excitatory inputs influence not only Golgi cells but also GCs, the regulation of MF excitatory inputs alone to Golgi cells are superficially ineffective for altering E-I balance at the level of GCs. In vivo recording of synaptic currents has, however, demonstrated that Golgi cells and GCs could be separately activated by different MFs [58], making it possible for the presence or absence of Golgi cell specific excitatory MF inputs to alter the E-I balance in GCs as well. Thus, the activity balance among different groups of GCs could be controlled by affecting the acetylcholine release in the GCL or the Golgi cell activity.

Based on the massive number of GCs present, with each having only 3–4 synaptic contacts with MFs, the cerebellar network in the GCL has been theoretically considered to be suitable for pattern separation via sparse GC activity [59, 60]. In contrast, in vivo Ca^2+^ imaging in GCs has demonstrated dense GC activation in behaving animals [61–63], contradicting sparse coding theory. Although whether there is indeed sparse or dense activation of GCs is still under debate [64], our results raise a possibility that structural organization-associated heterogeneous functional properties of GCs may contribute to the effective separation of multimodal MF inputs, as computationally predicted [65]. In addition, the MF-GC input stream includes other types of heterogeneity, such as heterogeneity at the level of synaptic transmission from MFs [47] and heterogeneity in the velocity of action potential propagation in the PFs [26]. Combination of these various heterogeneities may increase the diversity of MF input representation in the ML or may oppositely compensate the compelling parts of the observed heterogeneities to smooth or even cancel out the variabilities inherent in the unique structural organization of PFs in the ML. It is interesting and important to understand how the individual or combined functional heterogeneities would affect the information coding through the MF-GC input stream. For future investigations, it will be necessary to develop experimental methods that would allow experimenters to equalize these specific heterogeneities.

## Methods

### Animals

All procedures involving mice were performed according to the guidelines of the Institutional Animal Care and Use Committee of Korea Institute of Science and Technology. Because of their nursing ability, ICR mice of both sexes were used in this study.

### AAV production and injection

AAV vectors with estimated titers of approximately 10^13^ vector genome copies per mL were produced as described previously [66]. AAV constructs were made by cloning in plasmids for AAV-GABRα6 that were used in our previous study [66]. The cDNA fragment for dT was obtained from AAV-CaMKIIa-GCaMP6f-P2A-nls-dTomato (Addgene, #51087) and the fragment of mTB was obtained from pBAD-mTagBFP2 (Addgene, #34632). To trigger expression of fluorescent protein in GCs with PFs located at deep (D-GCs), middle (M-GCs), or superficial (S-GCs) sublayer of the ML, AAV-GABRα6 (about 1.5 μl total) were stereotaxically injected into cerebellar lobe IV/V of the cerebellar vermis at P7, P9, or P13, respectively. When two groups of GCs were labeled, for the second injection, the hole made in the cranium for the first injection was reused. After the surgery, mice were kept on a heating pad until they recovered from the anesthesia and then were returned to their home cages.

### Slice preparation and Ca^2+^ imaging

Chemicals used were obtained from Sigma or Wako Pure Chemical Industries, unless otherwise specified. Slices were prepared as previously described [67]. Sagittal cerebellar slices (200 μm) were obtained from P22 to P30 mice subjected to stereotaxic AAV injection, and stored in extracellular solution (ACSF) containing the following (in mM): 125 NaCl, 2.5 KCl, 1.3 MgCl_2_, 2 CaCl_2_, 1.25 NaH_2_PO_4_, 26 NaHCO_3_, and 20 glucose. To load the cerebellar slices with OGB1, a Ca^2+^ indicator dye, slices were placed in the recording chamber, a glass pipette connected to pneumatic microinjector (Pneumatic PicoPump, World Precision Instruments) was filled with OGB1 acetoxymethyl ester (OGB1-AM, Thermo Fisher Scientific, O6807), lowered ~ 40 um into the GCL of the cerebellar slices, and then a bolus of OGB1-AM was ejected into the cerebellar slice at 10 psi for 5 min to locally label the GCL. Before imaging the slices, slices were incubated in the recording chamber for 30 min to allow continuous perfusion by extracellular ACSF to wash away the excess OGB1-AM, leaving only the internalized OGB1.

Live cell imaging of OGB1 was performed using a microscope (Nikon Eclipse FN1) equipped with a high N.A. water immersion objective CFI75 LWD 16X W, an iXon Ultra 897 camera (Andor) and NIS elements imaging software (Nikon). Cropped images (304 × 304 pixel, 16 bits deep) of the GCLs were taken at 220 Hz, before, during, and after the MF burst stimulation (100 Hz, 100 ms). A concentric bipolar electrode (FHC Inc.) was placed on the white matter to apply the MF stimulation. The timing of the MF stimulation was controlled by the trigger-out feature of NIS elements software.

To tease apart the individual contribution of ionotropic and metabotropic receptors at the MF-GC synapse on the observed Ca^2+^ responses in the GCs, several inhibitors were added to the extracellular ACSF before and during the imaging. Inhibitors used were bicuculline (#0131, 10 μM), AP5 (#0106, 100 μM), CNQX (#1045, 25 μM), 7Cl (#3697, 50 μM), CGP (#1245, 50 μM), DL-TBOA (#1223, 50 μM), SNAP (#1561, 50 μM), and SKF (#1081, 100 μM). All inhibitors were obtained from Tocris Bioscience.

### Analysis of the Ca^2+^ responses

Individual GCs were selected from the dT or mTB images by the spot detection function of NIS Elements. The GCs with the 20 highest dT or mTB intensity were considered dT- or mTB-positive GCs. For the experiments using a single AAV-GABRα6-dT injection, we first identified 100 GCs detectable by their dT intensity, which presumably include both dT fluorescence and autofluorescence, and the GCs with the 20 (60, in Additional file 1: Fig. S3) lowest dT intensity were considered dT-negative GCs (other GCs). The time course of OGB1 fluorescent intensity change was obtained from the individual GCs (see an example in Additional file 1: Fig. S5a), and was smoothed out by Savitsky-Golay filtering (F, Additional file 1: Fig. S5b). Because the fluorescence intensity exponentially decayed over time, probably due to photobleaching, the baseline fluorescence had to be adjusted. To estimate the baseline fluorescence (F_baseline_) change over time, a part of smoothed time course before and after MF stimulation-dependent Ca^2+^ increase was fitted with an exponential decay curve (Additional file 1: Fig. S5c). The percent ratio of fluorescence intensity to baseline level (ΔF/F_0_, Additional file 1: Fig. S5d) was calculated using the equation, ΔF/F_0_ = ((F − F_baseline_)/F_baseline_) × 100. The integration of fluorescence increases upon MF stimulation (∫ΔF/F_0_∙dt) was calculated to quantify the total Ca^2+^ increase over time. The resultant values of ∫ΔF/F_0_∙dt represent the Ca^2+^ responses in individual GCs. However, each slice would have different distribution of the Ca^2+^ responses due to different labeling conditions, stimulation efficacy, or addition of inhibitors. To standardize the slice to slice variability, a z-score of the Ca^2+^ responses (standardized ∫ΔF/F_0_∙dt) was calculated by the equation (∫ΔF/F_0_∙dt − μ) / σ, in which μ and σ are a sample mean and a sample standard deviation of ∫ΔF/F_0_∙dt of all GCs detected in a single slice, respectively. The z-score of each GC was thus basically regarded as its Ca^2+^ response, and the “Ca^2+^ responses” in figures indicate the standardized ∫ΔF/F_0_∙dt (z-score), unless otherwise specified. To estimate the effect size of difference in Ca^2+^ responses between different GC groups, averaged differences (a) and the averages of total Ca^2+^ increase (b) are also calculated by using the unstandardized ∫ΔF/F_0_∙dt, and their ratio (a/b × 100) was used as the estimation (Additional file 1: Table S1). In addition to amounts of Ca^2+^ increase, the time course profiles of the Ca^2+^ responses were quantified by detecting time to peak (from 0.5% rise to peak) and decay time constant obtained from a single exponential fit. We made a macro in OriginPro software (OriginLab) for the process from smoothing to obtaining the values of ∫ΔF/F_0_∙dt to automate the analysis.

To compare the Ca^2+^ responses in two different groups of GCs, the average of z-scores in each group was calculated in individual slices, and the averaged z-score of one group was subtracted from the averaged z-score of the other group. For slices with AAV-GABRα6-dT single injection, the averaged z-score of other GCs were subtracted from the averaged z-score of D-GCs, M-GCs, or S-GCs. For slices with AAV-GABRα6-dT and AAV-GABRα6-mTB double injection, the z-score of deeper GCs was subtracted from the z-score of more superficial GCs (M-D, S-M, or S-D). The subtracted z-scores thus show the difference in Ca^2+^ responses between the two groups of GCs. For Additional file 1: Fig. S1, unstandardized ∫ΔF/F_0_∙dt and unstandardized peak ΔF/F_0_ were used to calculate differences in the Ca^2+^ responses. To minimize the influence of noise and to omit the results obtained from the unhealthy slices, fluorescent intensity was also measured from the whole GCL, and results were discarded if the peak ΔF/F_0_ of the whole GCL was less than 0.7.

To compare the Ca^2+^ responses while minimizing the contaminating signals from out-of-focus GCs, we manually combed the images of OGB1 and dT, and selected pairs of neighboring dT-positive GCs and other GCs, both of which were in focus, among GCs identified by the spot detection function of NIS Elements. We then calculated the difference in their ∫ΔF/F_0_∙dt, and tested the significance of the differences obtained from several pairs.

To examine how the numbers of analyzed GCs affected results of our analysis, we compared the Ca^2+^ responses between randomly picked certain numbers of GC pairs by the following procedure:

- A number (n = 1–17) of GC pairs of dT-positive GCs and dT-negative GCs were randomly selected in a single slice, and the difference in Ca^2+^ responses (∫ΔF/F_0_∙dt) was then calculated in all pairs.
- Such random selection and calculation were performed in all the slices. As a result, differences in Ca^2+^ responses from N numbers (N = GC pairs sampled per slice (n) × number of slices) of pairs were obtained, and their average is plotted as a single circle in Additional file 1: Fig. S2b.
- This procedure was repeated 20 times with a certain n, so that 20 different data are plotted for each N.
- We further performed statistical tests for the specific cases of n = 1 or 17 pairs sampled per slice (Additional file 1: Fig. S2c and d).

### Immunohistochemistry, and analysis of ratios and distributions of labeled GCs

Primary and secondary antibodies used were mouse anti-Kv4.2 (Neuromab, 75-016, RRID: AB_2131945) and Alexa Fluor 647-conjugated anti-mouse IgG (Thermo Fisher Scientific, A21236), respectively. Mice subjected to AAV-GABRα6-GFP injection were anesthetized at P21–P23, and perfused transcardially with 4% paraformaldehyde (PFA) in 0.1 M sodium phosphate buffer (pH 7.4). The cerebellum was post-fixed with 2% PFA and sagittally sectioned (40 µm) using a vibrating microtome (Leica VT 1200S). Immunohistochemistry was performed by following a protocol described previously [66]. Single optical sections of confocal images were acquired by an A1R laser scanning confocal microscope (Nikon).

Numbers of mice used for the analyses were 3 or 4 per injection time point. For the image analyses, all GC somas, including both GFP labeled and unlabeled GC somas, were detected by utilizing the spot detection function of NIS Elements on the images of GCLs stained with a Kv4.2 antibody, which was used to visualize the GC membranes. The analyses of the GCL were performed on 2 highly magnified images (0.21 × 0.21 mm) per mouse, and their average was taken as representation of each mouse. GCs were determined to be GFP-positive if more than 80% of the detected somas were labeled with GFP. The percentages of GFP-positive GC somas among all GCs were calculated to estimate the expression percentages. The positional information of GFP-labeled GC somas along anterior–posterior axis was used for the analysis of GC soma distribution. To estimate the location of GFP-positive PF bundles, line scan profiles across the Purkinje cell layer and the ML were taken at 5 different areas per mouse, and their average was taken to be the representation of each mouse.

### Statistical analyses

All sample sizes applied to the statistical test are indicated in the figure legends. Statistical differences were determined by the paired sample t-test for examining the effects of AP5 and CNQX on Ca^2+^ responses in whole GCL, by the KS test for the comparison of cumulative distributions, by one-way ANOVA with the Fisher test for the comparison of time to peak and decay time constants in GC groups, and by Mann–Whitney test for the comparison of Ca^2+^ responses between the presence and absence of bicuculline. Under the hypothesis that the Ca^2+^ responses in two GC groups are equivalent, the difference in their Ca^2+^ responses is supposed to be equal to 0. To test whether the Ca^2+^ responses were different in two GC groups, we therefore used the one-sample Wilcoxon signed rank test and compared the differences in Ca^2+^ responses with test median 0. To test whether the inhibitors significantly affect the difference in Ca^2+^ responses, or the Ca^2+^ responses themselves, we also performed a one-way ANOVA, followed by the post-hoc Fisher test. In addition, a two-way ANOVA was used to compare group data, such as comparison between data calculated using the standardized ∫ΔF/F_0_∙dt and the standardized peak ΔF/F_0_. Data are presented as mean ± SEM. Image analyses for Ca^2+^ responses were performed using NIS Elements and OriginPro software, and those for immunohistochemical images were performed using NIS Elements and ImageJ (National Institutes of Health) software. The statistical analyses were performed using OriginPro software. Exact *p* values for the datasets in this study are provided in Additional file 1: Table S2.

## Supplementary Information

**Additional file 1:** **Fig. S1.** Consistent results of the difference between the dT-positive GCs and the other GCs in analyses using the unstandardized ∫ΔF/F_0_∙dt or the unstandardized peak ΔF/F_0_. **Fig. S2. **Analysis of neighboring GC pairs and the effects of sample numbers. **Fig. 3.** Time course profiles of the Ca^2+^ responses in the presence of bicuculline and analyses with 60 other GCs. **Fig. S4. **Comparison of decay time constants. **Fig. S5.** The flow chart of how to calculate the Ca^2+^ responses in individual GCs. **Table S1. **Estimation of the effect size based on the calculation of percentage of the difference in ∫ΔF/F_0_•dt between two GC groups in averaged total ∫ΔF/F_0_•dt upon MF stimulation. **Table S2.** *P* values and statistical tests

## Data Availability

All original data and plasmids used in this study are available upon reasonable request.

## Abbreviations

GC: Granule cell
MF: Mossy fiber
PF: Parallel fiber
ML: Molecular layer
AAV: Adeno-associated virus
GCL: Granule cell layer
dT: dTomato
mTB: mTagBFP2
GABA_A_R: γ-Aminobutyric acid type A receptor
GABA_B_R: γ-Aminobutyric acid type B receptor
NMDAR: N-methyl-d-aspartate receptor
AMPAR: α-Amino-3-hydroxy-5-methyl-4-isoxazolepropionic acid receptor
GABRα6: GABA type A receptor α6 subunit
OGB1: Oregon Green 488 BAPTA-1
CNQX: 6-Cyano-7-nitroquinoxaline-2,3-dione
7Cl: 7-Chlorokynurenic acid
CGP: CGP35348
DL-TBOA: DL-threo-β-Benzyloxyaspartic acid
SNAP: (S)-SNAP 5114
SKF: SKF 89976A
