# Supplementary material for: Projection-dependent heterogeneity of cerebellar granule cell calcium responses
Source: Mol Brain. 2021 Mar 31;14:63. doi: 10.1186/s13041-021-00773-y (PMC8011397; doi:10.1186/s13041-021-00773-y)
Supplement: Supplementary file 1 — Additional file 1: Fig. S1. Consistent results of the difference between the dT-positive GCs and the other GCs in analyses using the unstandardized ∫ΔF/F0∙dt or the unstandardized peak ΔF/F0. Fig. S2. Analysis of neighboring GC pairs and the effects of sample numbers. Fig. 3. Time course profiles of the Ca2+ responses in the presence of bicuculline and analyses with 60 other GCs. Fig. S4. Comparison of decay time constants. Fig. S5. The flow chart of how to calculate the Ca2+ responses in individual GCs. Table S1. Estimation of the effect size based on the calculation of percentage of the difference in ∫ΔF/F0•dt between two GC groups in averaged total ∫ΔF/F0•dt upon MF stimulation. Table S2. P values and statistical tests [file 13041_2021_773_MOESM1_ESM.pdf]

Additional file 1:
Figure S1.
Figure S2.
Figure S3.
Figure S4.
Figure S5.
Table S1.
Table S2

Projection-dependent heterogeneity of cerebellar granule cell calcium responses

Jun Kyu Rhee, Heeyoun Park, Taegon Kim, Yukio Yamamoto, and Keiko Tanaka-Yamamoto

Figure S1

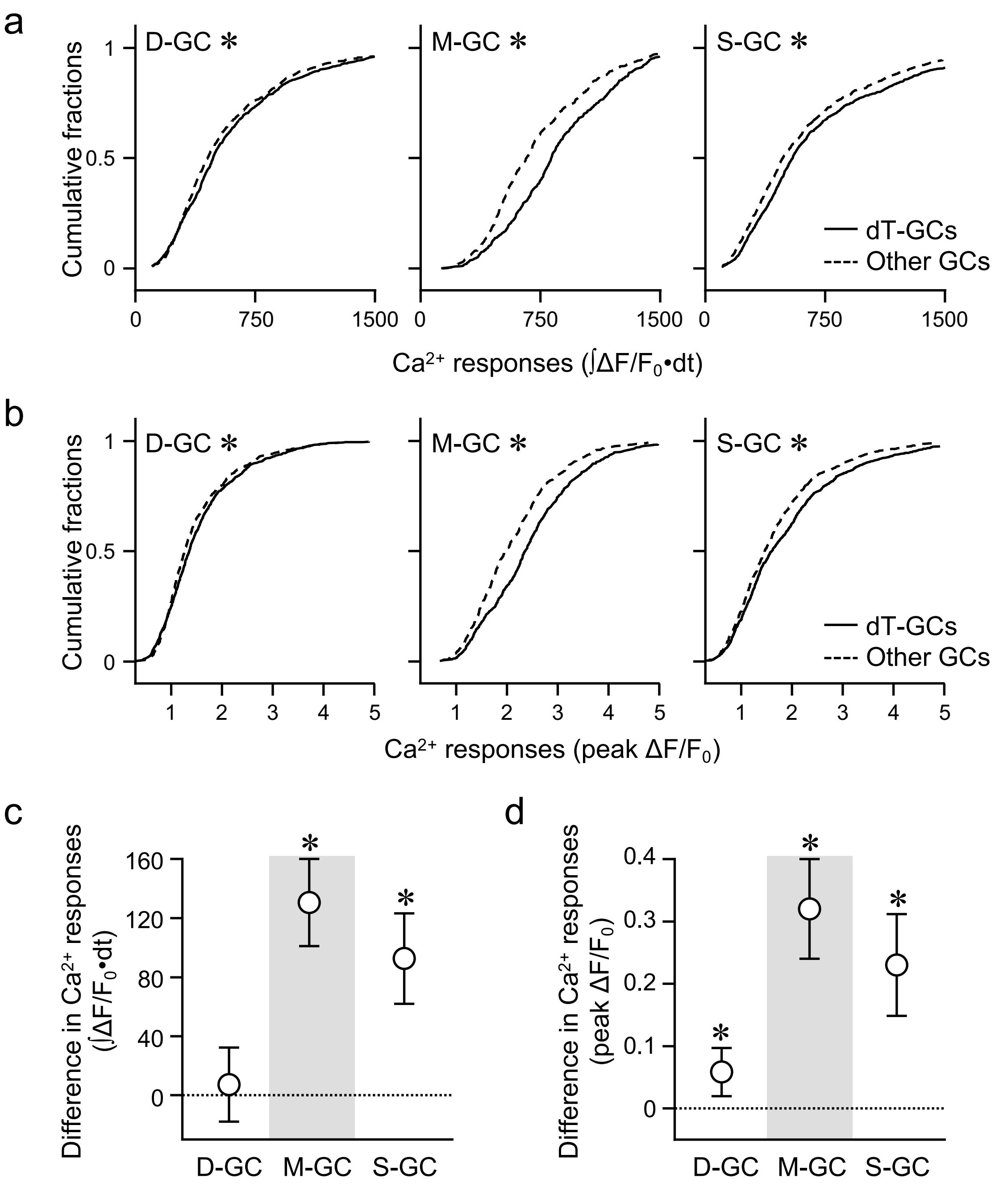

Fig. S1 Consistent results of the difference between the dT-positive GCs and the other GCs in analyses using the unstandardized $\int\Delta F/F_0 \cdot dt$ or the unstandardized peak $\Delta F/F_0$. **a, b** The cumulative distributions of the unstandardized $\int\Delta F/F_0 \cdot dt$ (**a**) or the unstandardized peak $\Delta F/F_0$ (**b**) in D-GCs, M-GCs, and S-GCs (solid lines; n = 1060, 620, or 1060 cells for D-GCs, M-GCs, or S-GCs, respectively) compared with the distributions in other GCs (dotted lines; n = 1060, 620, or 1060 cells for D-GCs, M-GCs, or S-GCs, respectively). **c, d** Difference in Ca^{2+} responses, represented by the unstandardized $\int\Delta F/F_0 \cdot dt$ (**c**) or the unstandardized peak $\Delta F/F_0$ (**d**), between dT-positive GCs (D-GCs, M-GCs, or S-GCs) and other GCs in control (black open circles; n = 53, 31, or 53 slices for D-GCs, M-GCs, or S-GCs, respectively; for the comparison with 0, *$p < 0.05$, one-sample Wilcoxon signed rank test).

Figure S2

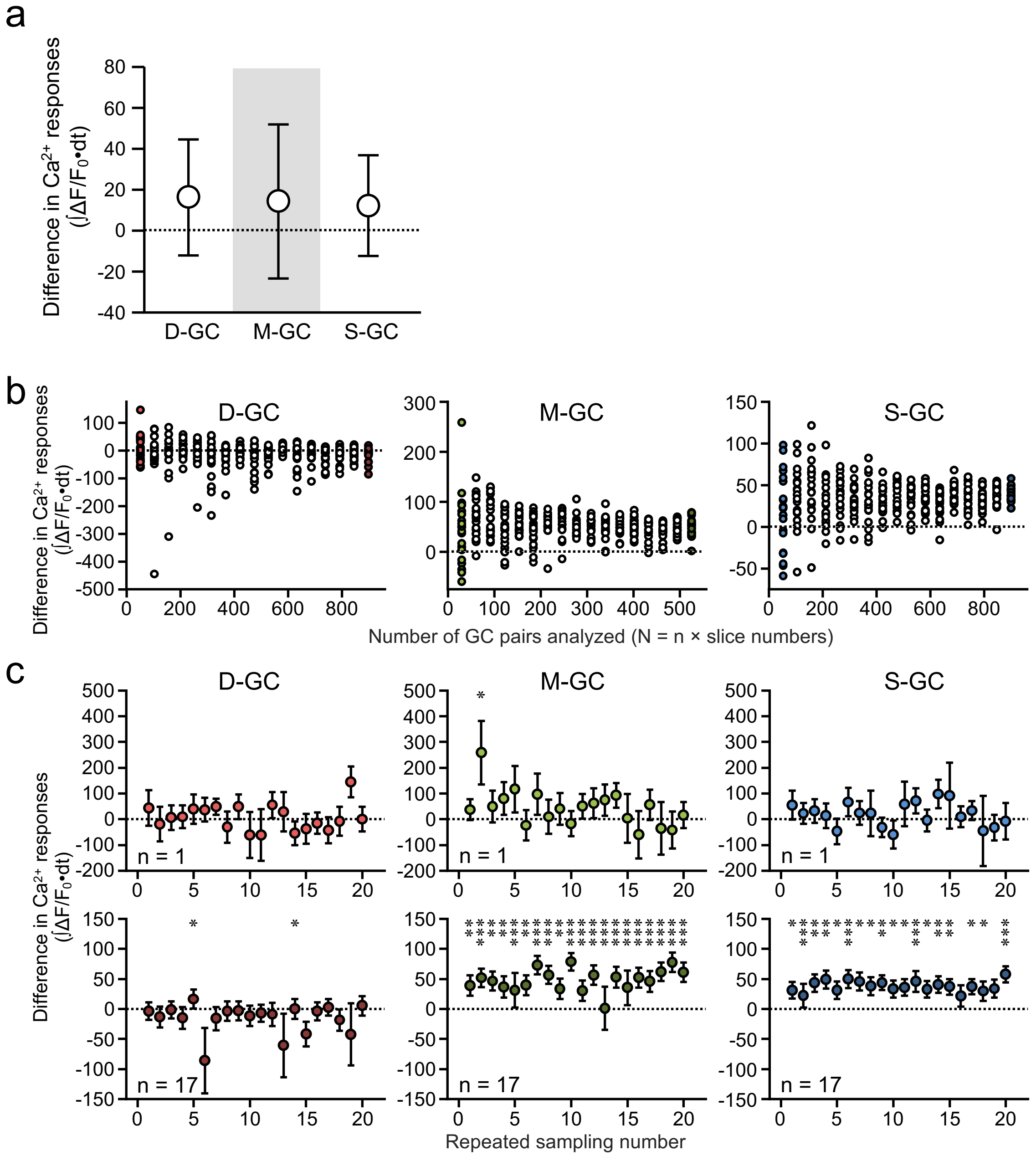

d

Repeated sampling number	n = 1 pair sampled per slice						n = 17 pairs sampled per slice					
	D-GC		M-GC		S-GC		D-GC		M-GC		S-GC	
1	1		0.347		0.337		0.422		0.00315	**	0.0392	*
2	0.46		0.0132	*	0.725		0.721		3.43×10^{-4}	***	4.72×10^{-4}	***
3	0.46		0.189		0.706		0.418		0.00105	**	0.0095	**
4	0.958		0.182		0.648		0.235		0.0099	**	0.00719	**
5	0.58		0.224		0.772		0.0355	*	9.75×10^{-5}	***	0.0171	*
6	0.586		0.529		0.623		0.992		0.00395	**	5.95×10^{-4}	***
7	0.365		0.357		0.316		0.222		2.05×10^{-7}	***	0.0135	*
8	0.429		0.931		0.923		0.327		5.74×10^{-5}	***	0.0107	*
9	0.263		0.399		0.568		0.574		0.0011	**	0.00178	**
10	0.972		0.542		0.574		0.862		2.65×10^{-8}	***	0.0302	*
11	0.32		0.239		0.466		0.662		2.65×10^{-4}	***	0.0157	*
12	0.648		0.378		0.075		0.643		4.32×10^{-6}	***	4.25×10^{-4}	***
13	0.909		0.0942		0.93		0.344		4.74×10^{-4}	***	0.0204	*
14	0.365		0.176		0.143		0.0257	*	1.85×10^{-4}	***	0.00264	**
15	0.429		0.794		0.106		0.823		4.71×10^{-5}	***	0.00126	**
16	0.979		0.622		0.819		0.305		3.92×10^{-5}	***	0.0889	
17	0.895		0.0502		0.499		0.653		7.74×10^{-4}	***	0.0213	*
18	0.346		0.664		0.155		0.521		4.92×10^{-6}	***	0.0289	*
19	0.112		0.678		0.493		0.0741		3.78×10^{-7}	***	0.0509	
20	0.868		0.7665		0.586		0.091		2.05×10^{-5}	***	3.37×10^{-6}	***

Fig. S2 Analysis of neighboring GC pairs and the effects of sample numbers.
a Difference in $\int \Delta F/F_0 \cdot dt$ between neighboring GC pairs of dT-positive GCs (D-GCs, M-GCs, or S-GCs) and other GCs in control (n = 50, 31, or 53 GC pairs for D-GCs, M-GCs, or S-GCs, respectively. **b-d** Difference in $\int \Delta F/F_0 \cdot dt$ between randomly selected pairs of dT-positive GCs and other GCs in control. Averaged values of difference are plotted against numbers (N = GC pairs sampled per slice (n) × number of slices) of pairs used for the analysis (**b**), which was repeated 20 times with a certain n. Data shown in color (n = 1 and 17) are replotted in **c**, which are shown as average values with SEM. In addition, *p* values of individual data shown in **c** are listed in **d** (for comparison with 0, *$p < 0.05$, **$p < 0.01$, ***$p < 0.001$, one-sample Wilcoxon signed rank test).

Figure S3

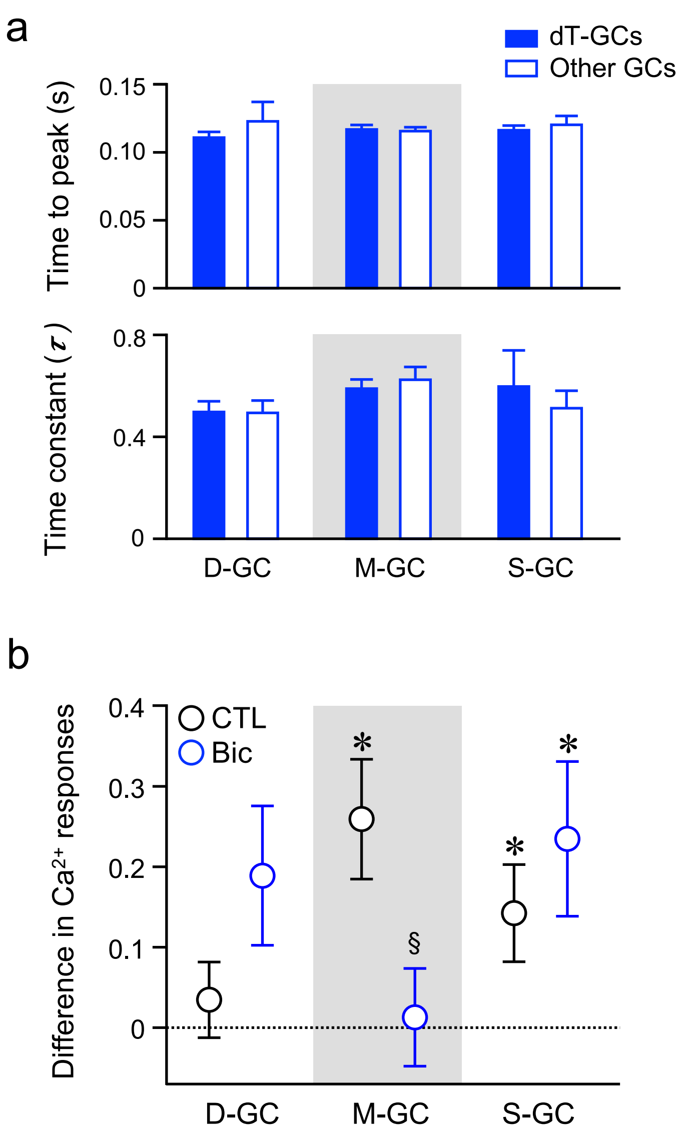

Fig. S3 Time course profiles of the Ca^{2+} responses in the presence of bicuculline and analyses with 60 other GCs.

a Comparison of time to peak and the decay time constants of the Ca^{2+} responses between dT-positive GCs (D-GCs, M-GCs or S-GCs) and other GCs in the presence of bicuculline (n = 27, 39, or 25 slices for D-GCs, M-GCs, or S-GCs, respectively). **b** Difference in Ca^{2+} responses between dT-positive GCs and 60 other GCs in control (black open circles; n = 53, 31, or 53 slices for D-GCs, M-GCs, or S-GCs, respectively) or in the presence of bicuculline (blue open circles; n = 27, 39, or 26 slices for D-GCs, M-GCs, or S-GCs, respectively) (for the comparison with 0, *p < 0.05, one-sample Wilcoxon signed rank test; for comparison between control and bicuculline, $^{§}p < 0.05$, one-way ANOVA followed by the Fisher test).

Figure S4

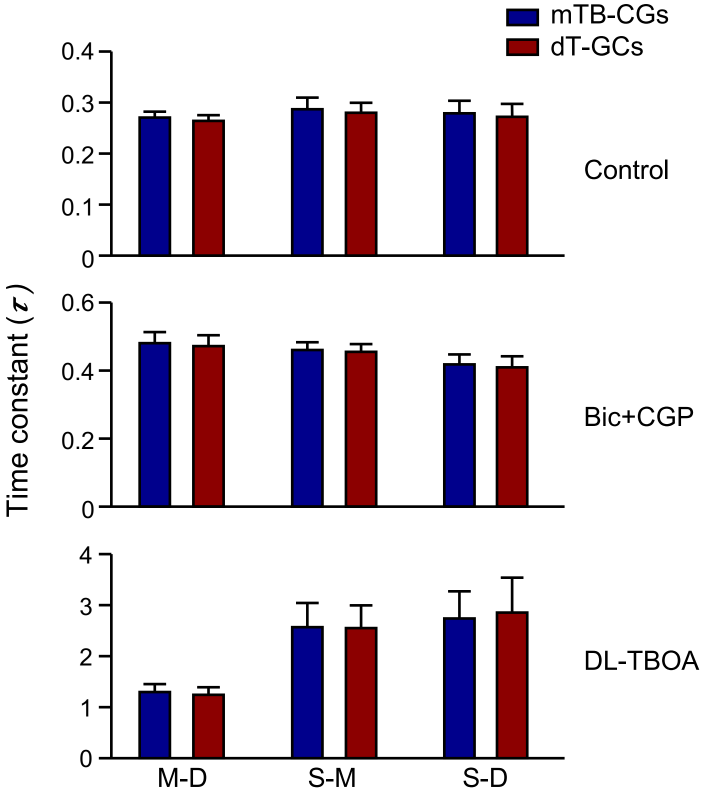

Fig. S4 Comparison of decay time constants.
Comparison of the decay time constants of the Ca^{2+} responses between mTB-positive GCs and dT-positive GCs in control (top, n = 32, 33, or 27 slices for M–D, S–M, or S–D, respectively), in the presence of bicuculline and CGP (middle, n = 19, 18, or 18 for M–D, S–M, or S–D, respectively), or in the presence of DL-TBOA (bottom, n = 19, 12, or 10 for M–D, S–M, or S–D, respectively).

Figure S5

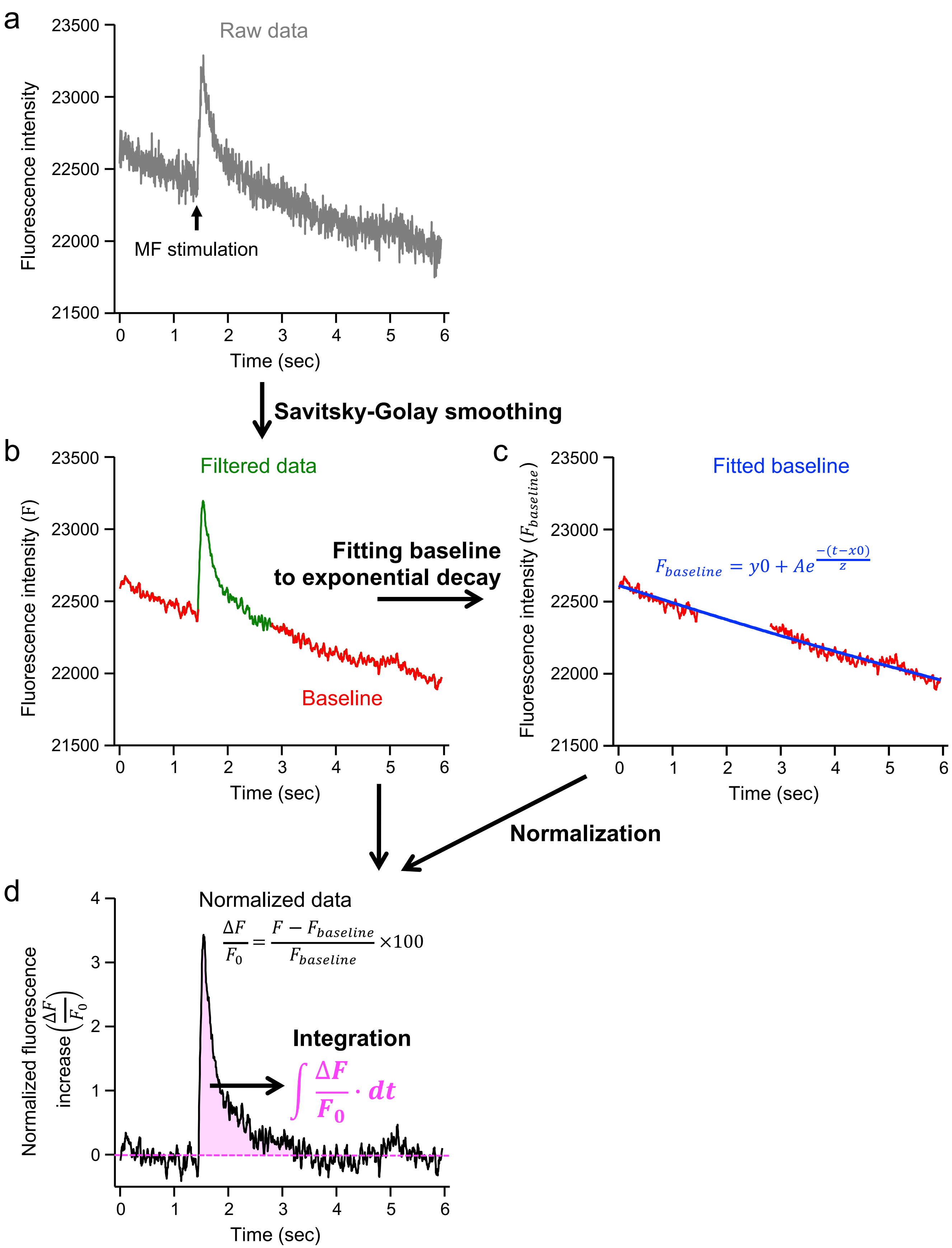
a
Raw data
Fluorescence intensity
23500
23000
22500
22000
21500
MF stimulation
0 1 2 3 4 5 6
Time (sec)
Savitsky-Golay smoothing
b
Filtered data
Fluorescence intensity (F)
Baseline
Time (sec)
Fitting baseline
to exponential decay
c
Fitted baseline
Fluorescence intensity ($F_{baseline}$)
$F_{baseline} = y0 + Ae^{\frac{-(t-x0)}{z}}$
Time (sec)
Normalization
d
Normalized data
$\frac{\Delta F}{F_0} = \frac{F - F_{baseline}}{F_{baseline}} \times 100$
Normalized fluorescence increase $\left(\frac{\Delta F}{F_0}\right)$
4
3
2
1
0
Integration
$\int \frac{\Delta F}{F_0} \cdot dt$
Time (sec)

Fig. S5 The flow chart of how to calculate the Ca^{2+} responses in individual GCs.
a Raw data (gray) obtained by measuring the fluorescence intensity of OGB1 over time. **b** Filtered time course (green) was obtained by smoothing the raw data using Savitsky-Golay filtering. A part of time course before and after the MF stimulation-dependent Ca^{2+} increase (red) was used to estimate the baseline. **c** The baseline was estimated by fitting the part of time course with an exponential decay function (blue). **d** The normalized time course was calculated using an equation, $\Delta F/F = (F-F_{baseline})/F_{baseline} \times 100$, and the Ca^{2+} responses were calculated as $\int \Delta F/F_0 \cdot dt$. A data example shown in **a** is used in **b**-**d**.

Table S1: Estimation of the effect size based on the calculation of percentage of the difference in ∫ΔF/F0•dt between two GC groups in averaged total ∫ΔF/F0•dt upon MF stimulation

Labeling		Inhibitors	Difference in Ca^{2+} responses (∫ΔF/F0•dt) (a)	*P*-values and significance§		Average of total Ca^{2+} increase in all GCs (∫ΔF/F0•dt) (b)	Estimated % of the difference in Ca^{2+} responses§§
Single/ Double	Compared GC groups						
Single	D-GCs & others	–	7.2 ± 25.1	0.158		609.5 ± 11.3	1.18
	M-GCs & others	–	130.6 ± 29.6	1.07×10^{-4}	***	802.7 ± 10.6	16.27
	S-GCs & others	–	92.6 ± 30.6	9.09×10^{-4}	***	677.9 ± 12.1	13.66
	D-GCs & others	Bicuculline	-14.3 ± 129.2	0.0619		1314.1 ± 70.7	1.09
	M-GCs & others	Bicuculline	27.1 ± 141.0	0.521		2928.1 ± 100.9	0.92
	S-GCs & others	Bicuculline	444.8 ± 212.1	0.014	*	1873.7 ± 92.3	23.74
Double	M-GCs & D-GCs	–	32.7 ± 16.0	0.028	*	934.6 ± 17.9	3.49
	S-GCs & M-GCs	–	2.3 ± 21.9	0.646		840.1 ± 16.0	0.28
	S-GCs & D-GCs	–	22.6 ± 22.8	0.135		937.0 ± 21.7	2.41
	M-GCs & D-GCs	Bicuculline	-31.3 ± 88.2	0.517		6824.9 ± 325.4	0.46
	S-GCs & M-GCs	Bicuculline	-25.0 ± 188.4	0.58		3419.0 ± 167.8	0.73
	S-GCs & D-GCs	Bicuculline	-126.9 ± 105.2	0.625		4826.6 ± 292.0	2.63
	M-GCs & D-GCs	Bic+CGP+AP5+7Cl	21.1 ± 17.2	0.266		693.8 ± 23.1	3.04
	S-GCs & M-GCs	Bic+CGP+AP5+7Cl	2.5 ± 16.6	0.702		879.9 ± 21.5	0.29
	S-GCs & D-GCs	Bic+CGP+AP5+7Cl	31.4 ± 26.6	0.623		976.5 ± 29.8	3.22
	M-GCs & D-GCs	Bic+CGP	208.6 ± 101.8	0.0258	*	4095.2 ± 129.7	5.09
	S-GCs & M-GCs	Bic+CGP	91.5 ± 57.1	0.13		2065.1 ±66.2	4.43
	S-GCs & D-GCs	Bic+CGP	70.1 ± 78.0	0.154		2563.2 ± 110.8	2.73
	M-GCs & D-GCs	DL-TBOA	512.9 ± 162.3	0.0024	**	8431.4 ± 375.7	6.08
	S-GCs & M-GCs	DL-TBOA	658.3 ± 725.6	0.176		32555.3 ± 1838.9	2.02
	S-GCs & D-GCs	DL-TBOA§§§	3503.8 ± 2030.4	0.131	(*)	36593.0 ± 3188.3	9.58
	M-GCs & D-GCs	CGP	-30.6 ± 21.2	0.291		1327.9 ± 24.3	2.3
	S-GCs & M-GCs	CGP	-2.7 ± 39.2	0.685		1073.1 ± 33.7	0.25
	S-GCs & D-GCs	CGP	-17.9 ± 21.6	0.469		584.7 ± 20.5	3.07
	M-GCs & D-GCs	SNAP+SKF	-73.3 ± 28.4	0.0219	*	1092.4 ± 33.3	6.71
	S-GCs & M-GCs	SNAP+SKF	103.0 ± 59.9	0.0107	*	2197.1 ± 92.0	4.69
	S-GCs & D-GCs	SNAP+SKF	89.1 ± 30.7	0.0134	*	1018.2 ± 27.7	8.75
	M-GCs & D-GCs	Bic+AP5+7Cl	18.2 ± 32.2	0.594		1056.8 ± 22.3	1.72
	S-GCs & M-GCs	Bic+AP5+7Cl	5.5 ± 50.5	0.664		1816.0 ± 48.8	0.3
	S-GCs & D-GCs	Bic+AP5+7Cl	47.3 ± 43.9	0.329		923.8 ± 39.1	5.12

§ Statistical test was performed by one sample Wilcoxon S-R test that examines significant difference with test median 0 (* $p < 0.05$, ** $p < 0.01$, *** $p < 0.001$).

§§ Percentage of the difference = |mean of (a)/mean of (b)| × 100

§§§ In case of comparison between S-GCs and M-GCs in the presence of DL-TBOA, significant difference was detected when standardized ∫ΔF/F0•dt was used for statistical test, despite no significant difference in unstandardized ∫ΔF/F0•dt.

Table S2: *P* values and statistical tests

Figures	Conditions/ Groups	Pair compared			*P* value	Significance§	Test
**Fig. 1d**	CNQX&AP5	Before	After		2.06 X 10^{-4}	***	Paired sample t-test
**Fig. 2d**		D-GCs	M-GCs	S-GCs	0.574		One-way ANOVA
**Fig. 3b**	Control (Time to peak)	D-GCs, M-GCs, S-GCs, and their other GCs			0.158		One-way ANOVA
		D-GCs	Other GCs		0.916		Fisher test
		M-GCs	Other GCs		0.882		Fisher test
		S-GCs	Other GCs		0.955		Fisher test
	Control (Decay time constant)	D-GCs, M-GCs, S-GCs, and their other GCs			0.587		One-way ANOVA
		D-GCs	Other GCs		0.899		Fisher test
		M-GCs	Other GCs		0.653		Fisher test
		S-GCs	Other GCs		0.837		Fisher test
**Fig. 3c**	Control (Standardized ∫ΔF/F0•dt)	D-GCs	Other GCs		0.0098	**	KS test
		M-GCs	Other GCs		7.32 X 10^{-23}	***	KS test
		S-GCs	Other GCs		6.93 X 10^{-19}	***	KS test
**Fig. 3d**	Control (Standardized ∫ΔF/F0•dt)	D-GCs	(Test median 0)		0.118		Wilcoxon S-R test
		M-GCs	(Test median 0)		1.07 X 10^{-4}	***	Wilcoxon S-R test
		S-GCs	(Test median 0)		6.16 X 10^{-4}	***	Wilcoxon S-R test
	Control (Standardized peak ΔF/F0)	D-GCs	(Test median 0)		0.0384	*	Wilcoxon S-R test
		M-GCs	(Test median 0)		1.31 X 10^{-4}	***	Wilcoxon S-R test
		S-GCs	(Test median 0)		0.00495	**	Wilcoxon S-R test
	Bicuculline (Standardized ∫ΔF/F0•dt)	D-GCs	(Test median 0)		0.0731		Wilcoxon S-R test
		M-GCs	(Test median 0)		0.799		Wilcoxon S-R test
		S-GCs	(Test median 0)		0.019	*	Wilcoxon S-R test
	Bicuculline (Standardized peak ΔF/F0)	D-GCs	(Test median 0)		0.0585		Wilcoxon S-R test
		M-GCs	(Test median 0)		0.167		Wilcoxon S-R test
		S-GCs	(Test median 0)		0.0176	*	Wilcoxon S-R test
	Multiple comparison of standardized ∫ΔF/F0•dt	D-GCs , M-GCs, and S-GCs with or without bicuculline			0.00508	**	One-way ANOVA
		D-GCs (CTL)	D-GCs (Bic)		0.294		Fisher test
		M-GCs (CTL)	M-GCs (Bic)		5.8 X 10^{-4}	***	Fisher test
		S-GCs (CTL)	S-GCs (Bic)		0.71		Fisher test
		All (CTL)	All (Bic)		0.252		Two-way ANOVA
	Multiple comparison of standardized peak ΔF/F0	D-GCs , M-GCs, and S-GCs with or without bicuculline			0.0514		One-way ANOVA
		D-GCs (CTL)	D-GCs (Bic)		0.331		Fisher test
		M-GCs (CTL)	M-GCs (Bic)		0.0103	*	Fisher test
		S-GCs (CTL)	S-GCs (Bic)		0.343		Fisher test
		All (CTL)	All (Bic)		0.718		Two-way ANOVA

§ Significance, * $p < 0.05$, ** $p < 0.01$, *** $p < 0.001$.

Table S2 (continued)

Figures	Conditions/ Groups	Pair compared		*P* value	Significance§	Test
**Fig. 3d** (Continued)	Control	Standardized ∫ΔF/F0•dt	Standardized peak ΔF/F0	0.642		Two-way ANOVA
	Bicuculline	Standardized ∫ΔF/F0•dt	Standardized peak ΔF/F0	0.305		Two-way ANOVA
**Fig. 3e**		Control	Bicuculline	0	***	Mann-Whitney test
**Fig. 3f**	Bicuculline (Standardized ∫ΔF/F0•dt)	D-GCs	Other GCs	7.05×10^{-7}	***	KS test
		M-GCs	Other GCs	0.0204	*	KS test
		S-GCs	Other GCs	1.96×10^{-14}	***	KS test
**Fig. 4b, 4c, 5b, 5c, 5d**	Control (Standardized ∫ΔF/F0•dt)	M-D	(Test median 0)	0.00934	**	Wilcoxon S-R test
		S-M	(Test median 0)	0.621		Wilcoxon S-R test
		S-D	(Test median 0)	0.0859		Wilcoxon S-R test
	Bicuculline (Standardized ∫ΔF/F0•dt)	M-D	(Test median 0)	0.342		Wilcoxon S-R test
		S-M	(Test median 0)	0.609		Wilcoxon S-R test
		S-D	(Test median 0)	0.232		Wilcoxon S-R test
	Bic+CGP+AP5+ 7Cl (Standardized ∫ΔF/F0•dt)	M-D	(Test median 0)	0.339		Wilcoxon S-R test
		S-M	(Test median 0)	0.304		Wilcoxon S-R test
		S-D	(Test median 0)	0.568		Wilcoxon S-R test
	Bic+CGP (Standardized ∫ΔF/F0•dt)	M-D	(Test median 0)	0.0494	*	Wilcoxon S-R test
		S-M	(Test median 0)	0.0814		Wilcoxon S-R test
		S-D	(Test median 0)	0.0987		Wilcoxon S-R test
	CGP (Standardized ∫ΔF/F0•dt)	M-D	(Test median 0)	0.515		Wilcoxon S-R test
		S-M	(Test median 0)	0.635		Wilcoxon S-R test
		S-D	(Test median 0)	0.469		Wilcoxon S-R test
	SNAP+SKF (Standardized ∫ΔF/F0•dt)	M-D	(Test median 0)	0.0393	*	Wilcoxon S-R test
		S-M	(Test median 0)	0.0134	*	Wilcoxon S-R test
		S-D	(Test median 0)	0.0134	*	Wilcoxon S-R test
	DL-TBOA (Standardized ∫ΔF/F0•dt)	M-D	(Test median 0)	2.67×10^{-4}	***	Wilcoxon S-R test
		S-M	(Test median 0)	0.129		Wilcoxon S-R test
		S-D	(Test median 0)	0.0371	*	Wilcoxon S-R test
	Bic+AP5+7Cl (Standardized ∫ΔF/F0•dt)	M-D	(Test median 0)	0.4		Wilcoxon S-R test
		S-M	(Test median 0)	0.839		Wilcoxon S-R test
		S-D	(Test median 0)	0.0858		Wilcoxon S-R test
	Multiple comparison	M-D (8 different conditions with or without inhibitors)		9.05×10^{-5}	***	One-way ANOVA
	M-D	Control	Bicuculline	3.22×10^{-4}	***	Fisher test
		Control	Bic+CGP+AP5+7Cl	0.481		Fisher test
		Control	Bic+CGP	0.685		Fisher test
		Control	CGP	0.011	*	Fisher test
		Control	SNAP+SKF	0.00103	**	Fisher test
		Control	DL-TBOA	0.319		Fisher test

§ Significance, * $p < 0.05$, ** $p < 0.01$, *** $p < 0.001$.

Table S2 (continued)

Figures	Conditions/ Groups	Pair compared		*P* value	Significance§	Test
Fig. 4b, 4c, 5b, 5c, 5d (Continued)	M-D	Control	Bic+AP5+7Cl	0.151		Fisher test
		Bicuculline	Bic+CGP+AP5+7Cl	0.0233	*	Fisher test
		Bicuculline	Bic+CGP	0.00364	**	Fisher test
		Bicuculline	CGP	0.177		Fisher test
		Bicuculline	SNAP+SKF	0.504		Fisher test
		Bicuculline	DL-TBOA	4.81×10^{-5}	***	Fisher test
		Bicuculline	Bic+AP5+7Cl	0.0203	*	Fisher test
		Bic+CGP+AP5+7Cl	Bic+CGP	0.743		Fisher test
		Bic+CGP+AP5+7Cl	CGP	0.209		Fisher test
		Bic+CGP+AP5+7Cl	SNAP+SKF	0.0647		Fisher test
		Bic+CGP+AP5+7Cl	DL-TBOA	0.153		Fisher test
		Bic+CGP+AP5+7Cl	Bic+AP5+7Cl	0.692		Fisher test
		Bic+CGP	CGP	0.0648		Fisher test
		Bic+CGP	SNAP+SKF	0.0119	*	Fisher test
		Bic+CGP	DL-TBOA	0.209		Fisher test
		Bic+CGP	Bic+AP5+7Cl	0.389		Fisher test
		CGP	SNAP+SKF	0.448		Fisher test
		CGP	DL-TBOA	0.00157	**	Fisher test
		CGP	Bic+AP5+7Cl	0.273		Fisher test
		SNAP+SKF	DL-TBOA	1.46×10^{-4}	***	Fisher test
		SNAP+SKF	Bic+AP5+7Cl	0.0651		Fisher test
		DL-TBOA	Bic+AP5+7Cl	0.0272	*	Fisher test
	Multiple comparison	S-M (8 different conditions with or without inhibitors)		0.825		One-way ANOVA
	S-M	Control	Bicuculline	0.69		Fisher test
		Control	Bic+CGP+AP5+7Cl	0.478		Fisher test
		Control	Bic+CGP	0.238		Fisher test
		Control	CGP	0.809		Fisher test
		Control	SNAP+SKF	0.312		Fisher test
		Control	DL-TBOA	0.28		Fisher test
		Control	Bic+AP5+7Cl	0.891		Fisher test
		Bicuculline	Bic+CGP+AP5+7Cl	0.784		Fisher test
		Bicuculline	Bic+CGP	0.491		Fisher test
		Bicuculline	CGP	0.918		Fisher test
		Bicuculline	SNAP+SKF	0.563		Fisher test
		Bicuculline	DL-TBOA	0.506		Fisher test
		Bicuculline	Bic+AP5+7Cl	0.611		Fisher test
		Bic+CGP+AP5+7Cl	Bic+CGP	0.678		Fisher test
		Bic+CGP+AP5+7Cl	CGP	0.724		Fisher test

§ Significance, * $p < 0.05$, ** $p < 0.01$, *** $p < 0.001$.

Table S2 (continued)

Figures	Conditions/ Groups	Pair compared		*P* value	Significance§	Test
**Fig. 4b, 4c, 5b, 5c, 5d** (Continued)	S-M	Bic+CGP+AP5+7Cl	SNAP+SKF	0.747		Fisher test
		Bic+CGP+AP5+7Cl	DL-TBOA	0.674		Fisher test
		Bic+CGP+AP5+7Cl	Bic+AP5+7Cl	0.414		Fisher test
		Bic+CGP	CGP	0.463		Fisher test
		Bic+CGP	SNAP+SKF	0.947		Fisher test
		Bic+CGP	DL-TBOA	0.961		Fisher test
		Bic+CGP	Bic+AP5+7Cl	0.2		Fisher test
		CGP	SNAP+SKF	0.527		Fisher test
		CGP	DL-TBOA	0.476		Fisher test
		CGP	Bic+AP5+7Cl	0.731		Fisher test
		SNAP+SKF	DL-TBOA	0.916		Fisher test
		SNAP+SKF	Bic+AP5+7Cl	0.268		Fisher test
		DL-TBOA	Bic+AP5+7Cl	0.242		Fisher test
	Multiple comparison	S-D (8 different conditions with or without inhibitors)		0.153		One-way ANOVA
	S-D	Control	Bicuculline	0.0944		Fisher test
		Control	Bic+CGP+AP5+7Cl	0.462		Fisher test
		Control	Bic+CGP	0.942		Fisher test
		Control	CGP	0.117		Fisher test
		Control	SNAP+SKF	0.537		Fisher test
		Control	DL-TBOA	0.175		Fisher test
		Control	Bic+AP5+7Cl	0.835		Fisher test
		Bicuculline	Bic+CGP+AP5+7Cl	0.304		Fisher test
		Bicuculline	Bic+CGP	0.104		Fisher test
		Bicuculline	CGP	0.927		Fisher test
		Bicuculline	SNAP+SKF	0.0477	*	Fisher test
		Bicuculline	DL-TBOA	0.013	*	Fisher test
		Bicuculline	Bic+AP5+7Cl	0.0737		Fisher test
		Bic+CGP+AP5+7Cl	Bic+CGP	0.462		Fisher test
		Bic+CGP+AP5+7Cl	CGP	0.313		Fisher test
		Bic+CGP+AP5+7Cl	SNAP+SKF	0.23		Fisher test
		Bic+CGP+AP5+7Cl	DL-TBOA	0.066		Fisher test
		Bic+CGP+AP5+7Cl	Bic+AP5+7Cl	0.368		Fisher test
		Bic+CGP	CGP	0.123		Fisher test
		Bic+CGP	SNAP+SKF	0.61		Fisher test
		Bic+CGP	DL-TBOA	0.224		Fisher test
		Bic+CGP	Bic+AP5+7Cl	0.906		Fisher test
		CGP	SNAP+SKF	0.0617		Fisher test
		CGP	DL-TBOA	0.0189	*	Fisher test

§ Significance, * $p < 0.05$, ** $p < 0.01$, *** $p < 0.001$.

Table S2 (continued)

Figures	Conditions/ Groups	Pair compared			*P* value	Significance§	Test
**Fig. 4b, 4c, 5b, 5c, 5d** (Continued)	S-D	CGP	Bic+AP5+7Cl		0.0941		Fisher test
		SNAP+SKF	DL-TBOA		0.47		Fisher test
		SNAP+SKF	Bic+AP5+7Cl		0.67		Fisher test
		DL-TBOA	Bic+AP5+7Cl		0.243		Fisher test
	Multiple comparison (with or without inhibitors)	M-D	S-M	S-D	0.269		Two-way ANOVA
	Multiple comparison (M-D, S-M, S-D)	8 different conditions with or without inhibitors			7.64×10^{-4}	***	Two-way ANOVA
	M-D<br>S-M<br>S-D	Control	Bicuculline		0.00419	**	Fisher test
		Control	Bic+CGP+AP5+7Cl		0.604		Fisher test
		Control	Bic+CGP		0.612		Fisher test
		Control	CGP		0.0247	*	Fisher test
		Control	SNAP+SKF		0.187		Fisher test
		Control	DL-TBOA		0.0322	*	Fisher test
		Control	Bic+AP5+7Cl		0.393		Fisher test
		Bicuculline	Bic+CGP+AP5+7Cl		0.037	*	Fisher test
		Bicuculline	Bic+CGP		0.00255	**	Fisher test
		Bicuculline	CGP		0.578		Fisher test
		Bicuculline	SNAP+SKF		0.145		Fisher test
		Bicuculline	DL-TBOA		2.15×10^{-5}	***	Fisher test
		Bicuculline	Bic+AP5+7Cl		0.0349	*	Fisher test
		Bic+CGP+AP5+7Cl	Bic+CGP		0.365		Fisher test
		Bic+CGP+AP5+7Cl	CGP		0.126		Fisher test
		Bic+CGP+AP5+7Cl	SNAP+SKF		0.493		Fisher test
		Bic+CGP+AP5+7Cl	DL-TBOA		0.0198	*	Fisher test
		Bic+CGP+AP5+7Cl	Bic+AP5+7Cl		0.832		Fisher test
		Bic+CGP	CGP		0.0139	*	Fisher test
		Bic+CGP	SNAP+SKF		0.103		Fisher test
		Bic+CGP	DL-TBOA		0.125		Fisher test
		Bic+CGP	Bic+AP5+7Cl		0.216		Fisher test
		CGP	SNAP+SKF		0.374		Fisher test
		CGP	DL-TBOA		1.83×10^{-4}	***	Fisher test
		CGP	Bic+AP5+7Cl		0.135		Fisher test
		SNAP+SKF	DL-TBOA		0.00255	**	Fisher test
		SNAP+SKF	Bic+AP5+7Cl		0.581		Fisher test
		DL-TBOA	Bic+AP5+7Cl		0.0056	**	Fisher test

§ Significance, * $p < 0.05$, ** $p < 0.01$, *** $p < 0.001$.

Table S2 (continued)

Figures	Conditions/ Groups	Pair compared		*P* value	Significance§	Test
Fig. 6a	Multiple comparison	∫ΔF/F0•dt (8 different conditions with or without inhibitors)		0	***	One-way ANOVA
	∫ΔF/F0•dt	Control	Bicuculline	2.26×10^{-30}	***	Fisher test
		Control	Bic+CGP+AP5+7Cl	0.932		Fisher test
		Control	Bic+CGP	1.65×10^{-9}	***	Fisher test
		Control	CGP	0.498		Fisher test
		Control	SNAP+SKF	0.173		Fisher test
		Control	DL-TBOA	0	***	Fisher test
		Control	Bic+AP5+7Cl	0.174		Fisher test
		Bicuculline	Bic+CGP+AP5+7Cl	2.77×10^{-24}	***	Fisher test
		Bicuculline	Bic+CGP	1.39×10^{-7}	***	Fisher test
		Bicuculline	CGP	7.97×10^{-21}	***	Fisher test
		Bicuculline	SNAP+SKF	6.98×10^{-20}	***	Fisher test
		Bicuculline	DL-TBOA	0	***	Fisher test
		Bicuculline	Bic+AP5+7Cl	5.57×10^{-24}	***	Fisher test
		Bic+CGP+AP5+7Cl	Bic+CGP	1.11×10^{-7}	***	Fisher test
		Bic+CGP+AP5+7Cl	CGP	0.503		Fisher test
		Bic+CGP+AP5+7Cl	SNAP+SKF	0.207		Fisher test
		Bic+CGP+AP5+7Cl	DL-TBOA	0	***	Fisher test
		Bic+CGP+AP5+7Cl	Bic+AP5+7Cl	0.22		Fisher test
		Bic+CGP	CGP	5.84×10^{-6}	***	Fisher test
		Bic+CGP	SNAP+SKF	3.78×10^{-5}	***	Fisher test
		Bic+CGP	DL-TBOA	0	***	Fisher test
		Bic+CGP	Bic+AP5+7Cl	2.62×10^{-6}	***	Fisher test
		CGP	SNAP+SKF	0.577		Fisher test
		CGP	DL-TBOA	0	***	Fisher test
		CGP	Bic+AP5+7Cl	0.647		Fisher test
		SNAP+SKF	DL-TBOA	0	***	Fisher test
		SNAP+SKF	Bic+AP5+7Cl	0.875		Fisher test
		DL-TBOA	Bic+AP5+7Cl	0	***	Fisher test

§ Significance, * $p < 0.05$, ** $p < 0.01$, *** $p < 0.001$.

Table S2 (continued)

Figures	Conditions/ Groups	Pair compared		*P* value	Significance§	Test
**Fig. S1a**	Control (∫ΔF/F0•dt)	D-GCs	Other GCs	0.00719	**	KS test
		M-GCs	Other GCs	6.3×10^{-13}	***	KS test
		S-GCs	Other GCs	0.00149	**	KS test
**Fig. S1b**	Control (Peak ΔF/F0)	D-GCs	Other GCs	0.0068	**	KS test
		M-GCs	Other GCs	7.74×10^{-9}	***	KS test
		S-GCs	Other GCs	1.36×10^{-4}	***	KS test
**Fig. S1c**	Control (∫ΔF/F0•dt)	D-GCs	(Test median 0)	0.158		Wilcoxon S-R test
		M-GCs	(Test median 0)	1.07×10^{-4}	***	Wilcoxon S-R test
		S-GCs	(Test median 0)	9.09×10^{-4}	***	Wilcoxon S-R test
**Fig. S1d**	Control (Peak ΔF/F0)	D-GCs	(Test median 0)	0.0468	*	Wilcoxon S-R test
		M-GCs	(Test median 0)	9.62×10^{-5}	***	Wilcoxon S-R test
		S-GCs	(Test median 0)	0.00197	**	Wilcoxon S-R test
**Fig. S2a**	Control (Comparison of neighboring GCs)	D-GCs	(Test median 0)	0.839		Wilcoxon S-R test
		M-GCs	(Test median 0)	0.915		Wilcoxon S-R test
		S-GCs	(Test median 0)	0.759		Wilcoxon S-R test
**Fig. S2c**	Control (Comparison of randomly sampled GCs)	See Figure S2d				
**Fig. S3a**	Bicuculline (Time to peak)	D-GCs, M-GCs, S-GCs, and their other GCs		0.864		One-way ANOVA
		D-GCs	Other GCs	0.212		Fisher test
		M-GCs	Other GCs	0.874		Fisher test
		S-GCs	Other GCs	0.679		Fisher test
	Bicuculline (Decay time constant)	D-GCs, M-GCs, S-GCs, and their other GCs		0.592		One-way ANOVA
		D-GCs	Other GCs	0.972		Fisher test
		M-GCs	Other GCs	0.672		Fisher test
		S-GCs	Other GCs	0.414		Fisher test
**Fig. S3b**	Control (Standardized ∫ΔF/F0•dt)	D-GCs	(Test median 0)	0.45		Wilcoxon S-R test
		M-GCs	(Test median 0)	0.0025	**	Wilcoxon S-R test
		S-GCs	(Test median 0)	0.0186	*	Wilcoxon S-R test
	Bicuculline (Standardized ∫ΔF/F0•dt)	D-GCs	(Test median 0)	0.692		Wilcoxon S-R test
		M-GCs	(Test median 0)	0.82		Wilcoxon S-R test
		S-GCs	(Test median 0)	0.022	*	Wilcoxon S-R test
	Multiple comparison of standardized ∫ΔF/F0•dt	D-GCs , M-GCs, and S-GCs with or without bicuculline		0.0504		One-way ANOVA
		D-GCs (CTL)	D-GCs (Bic)	0.116		Fisher test
		M-GCs (CTL)	M-GCs (Bic)	0.0139	*	Fisher test
		S-GCs (CTL)	S-GCs (Bic)	0.352		Fisher test
		All (CTL)	All (Bic)	0.971		Two-way ANOVA

§ Significance, * $p < 0.05$, ** $p < 0.01$, *** $p < 0.001$.

Table S2 (continued)

Figures	Conditions/ Groups	Pair compared		*P* value	Significance§	Test
Fig. S4	Control (Decay time constant)	M-GCs and D-GCs in M-D, S-GCs and M-GCs in S-M, S-GCs and D-GCs in S-D		0.972		One-way ANOVA
		M-GCs	D-GCs	0.796		Fisher test
		S-GCs	M-SGc	0.799		Fisher test
		S-GCs	D-GCs	0.819		Fisher test
	DL-TBOA (Decay time constant)	M-GCs and D-GCs in M-D, S-GCs and M-GCs in S-M, S-GCs and D-GCs in S-D		7.77×10^{-4}		One-way ANOVA
		M-GCs	D-GCs	0.905		Fisher test
		S-GCs	M-SGc	0.973		Fisher test
		S-GCs	D-GCs	0.845		Fisher test
	Bic+CGP (Decay time constant)	M-GCs and D-GCs in M-D, S-GCs and M-GCs in S-M, S-GCs and D-GCs in S-D		0.396		One-way ANOVA
		M-GCs	D-GCs	0.817		Fisher test
		S-GCs	M-SGc	0.898		Fisher test
		S-GCs	D-GCs	0.83		Fisher test

§ Significance, * $p < 0.05$, ** $p < 0.01$, *** $p < 0.001$.
